# Supplementary material for: Rapid planning and analysis of high-throughput experiment arrays for reaction discovery
Source: Nat Commun. 2023 Jul 3;14:3924. doi: 10.1038/s41467-023-39531-0 (PMC10318092; doi:10.1038/s41467-023-39531-0)
Supplement: Supplementary file 1 — Supplementary Information [file 41467_2023_39531_MOESM1_ESM.pdf]

Supplementary Information

Rapid Planning and Analysis of High-Throughput Experiment Arrays for Reaction Discovery

Babak Mahjour[1], Rui Zhang[2], Yuning Shen[1], Andrew McGrath[1], Ruheng Zhao[1], Osama G. Mohamed[3], Yingfu Lin[1], Zirong Zhang[1], James L. Douthwaite[1], Ashootosh Tripathi[1,3], Tim Cernak*[1,2]

[1]Department of Medicinal Chemistry, University of Michigan
[2]Department of Chemistry, University of Michigan
[3]Natural Products Discovery Core, Life Sciences Institute, University of Michigan
*Email: tcernak@umich.edu

Table of Contents

A. Supplementary Discussion
- I. Walkthrough and Tutorial for phactor™
  - a. Overview
  - b. Tutorial
  - c. Provided Example Files and Screenshots
    - i. Esterification reaction array (*Figure 2a*)
    - ii. Indolization reaction array (*Figure* 2b)
    - iii. Amide Pilot reaction array (*Figure 3a*)
    - iv. 96-well amine acid sp^3-sp^3 C–C coupling (*Figure 3d*)
    - v. 96-well amine acid sp^3-sp^3 C–C coupling (*Figure 3e*)
    - vi. 96-well amine acid sp^3-sp^2 C–C coupling (*Figure 3f*)
  - d. Database
  - e. API Connectivity
    - i. Analytical Trends
    - ii. UV Difference Heatmap
    - iii. Flask API Endpoint Example
    - iv. React Button UI and API Calling Example
    - v. OpenTrons OT-2 Conversion API Example
  - f. Conversion Scripts
    - i. Open Reaction Database
    - ii. Chemical Description Language
    - iii. Experimental Design via Bayesian Optimization+

B. Supplementary Methods
- II. Experimental
  - a. General information
  - b. Inhibition Assay General Information
  - c. Selected Screening Examples
    - i. Furan Indolization Reaction Array Procedure
    - ii. Mannich Reaction Array Procedure
    - iii. Synthesis of 7-chlorohept-1-en-4-one (**15**)
    - iv. Synthesis of methyl 3-(6-methoxy-3-(2-methoxy-2-oxoethyl)quinolin-4-yl)propanoate (**17**)
    - v. sp^2–sp^3 decarboxylative deaminative C–C coupling (*Figure 3f*)
    - vi. ultraHTE Exploratory Amine Acid Aryl Esterification Procedure (*Figure 3g*)
  - d. M^{Pro} inhibitor discovery direct-to-biology with phactor™.
    - i. Pilot Amide Reaction Array Procedure (*Figure 3a*)
    - ii. M^{Pro} inhibition assay
    - iii. Amide Pilot Reaction Array Results and IC_{50} Results
    - iv. 1,280-well ultraHTE direct-to-biology Procedure (*Figure 4*)
    - v. Direct-to-biology Analysis
    - vi. Preparation of the Carboxylic Acid Pharmacophore Substrate
    - vii. Scale up of N-(benzo[d]thiazol-2-yl)-2-oxo-1,2,3,4-tetrahydroquinoline-4-carboxamide
    - viii. Scale up of N-(4-(tert-butyl)phenyl)-2-oxo-1,2,3,4-tetrahydroquinoline-4-carboxamide
    - ix. Scale up of 2-oxo-N-(*p*-tolyl)-1,2,3,4-tetrahydroquinoline-4-carboxamide
  - e. NMR Spectra

C. Supplementary References

Supplementary Discussion

I. Walkthrough and Tutorial for phactor™

a. Overview. We have provided phactor™ as a web service to the academic community to enable a standardized format of executing and reporting high throughput experiments. This service is planned to be supported indefinitely and is limited to size 24 and 96 wellplate experiments. Rolling updates will be made with respect to feedback and communal use. The workflow provided is completely transient and stores no data whatsoever. As such, the web service runs on comma separated files as inputs and outputs to execute the workflow. Examples of these files are provided at https://github.com/cernaklab/public-phactor-example-files. These files contain all necessary information to reproduce

the 24 and 96 well experiments presented in this manuscript. Expected outputs from using these files are displayed in Supplementary Figures 2-7.

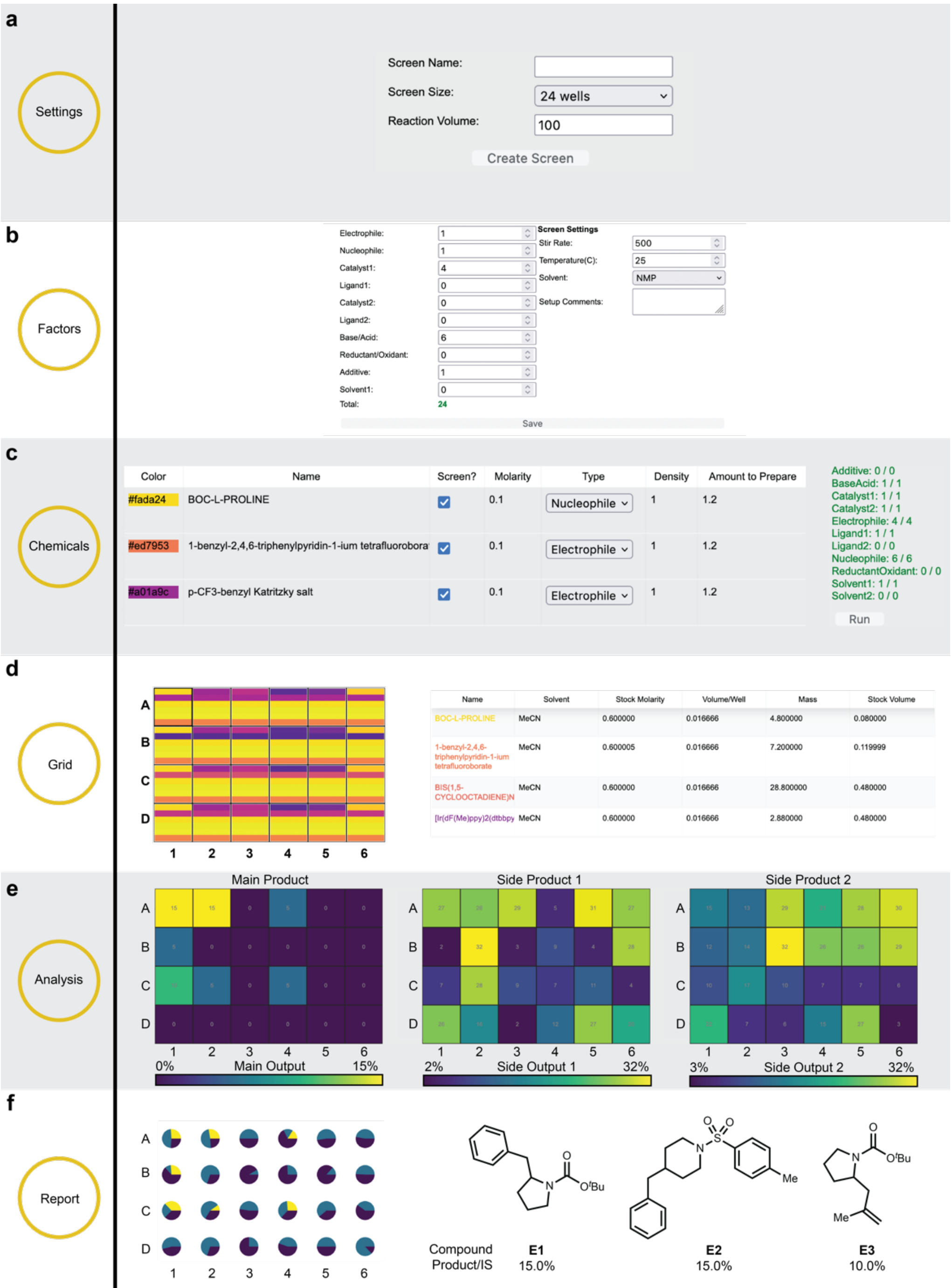

Supplementary Figure 1. Phactor™'s workflow consists of six stages: Settings, Factors, Chemicals, Grid, Analysis, and Report. With the correct files in hand, you can design a reaction array in seconds. **a)** To begin a reaction array design the user inputs a name for the experiment and specifies the plate throughput and reaction volume. **b)** Factors and metadata are input. The product of the factor values should be less than or equal to the plate throughput. **c)** The *Chemicals* stage allows for the manual addition of chemical species or compounds as well as direct input from embedded inventories. If the reagent labels match the specified factor distribution, the reaction array can be automatically populated. **d)** The *Grid* stage allows for the user to interactively edit the reaction array, as well as download the procedure to execute the experiment manually or via liquid-handling robot. Analysis setup files are provided at this stage as well. **e)** The *Analysis* stage expects an input file containing the location of each well and its corresponding characterized output value (generally UPLC-MS integrations). Three output displays can be shown in the same view. **f)** The *Report* stage summarizes the results of the experiment, providing visualizations and basic statistics regarding the performance of wells and reagents.

b. Tutorial. We begin the phactor™ HTE workflow by navigating to https://phactor.cernaklab.com. The landing page, **Settings**, displays terms of service and privacy policy associated with use of the software. This stage simply asks for a name for the experiment, its throughput (24 or 96 wells), and the desired reaction volume for each well (typically 100 μL at these throughputs). Clicking 'Create Screen' takes the user to the next stage, **Factors**.

The **Factors** stage is largely optional – inputting the experimental design in terms of reagent distributions allows for automated plate design. For instance, a 24 well experiment may screen 4 ligands and 6 catalysts. Inputting 4 and 6 into the respective textboxes indicates to the software that these reagents will be screened and should expect the corresponding chemicals to be added. Factors assigned 0 are ignored and factors assigned 1 are distributed into each well. As mentioned, this step is optional, and the reaction array can be designed entirely by hand in the **Grid** stage as desired.

Additionally, the **Factors** stage of the workflow expects input regarding experimental metadata and anticipated products. Stir rate, temperature, and solvent can be recorded here in addition to user defined commentary. Clicking the Set Products' button displays a popup with fields to describe expected products and side products. Currently, the expected product and two side products are supported. Each product or side product should be input with an associated SMILES and descriptive name. This form can be automatically populated with a csv with the following headers: [Well, main_product_name, main_product_smiles, side_product1_name, side_product1_smiles, side_product2_name, side_product2_smiles], where the Well column dictates the location of the products via well label (e.g., A1, D6, etc.). Examples and templates for this file can be found in the 'input_product_input' folder of the provided GitHub repository. Notably, this step is optional and is only necessary to automatically generate analytical files in the **Grid** stage. Clicking save records the information and takes the user to the **Chemicals** stage.

The **Chemicals** stage collects information regarding substrates and reagents planning to be used in the reaction array. Chemicals input here need not be included in the actual experiment. If screening factors were defined in the previous stage, the checklist in the bottom left indicates the amount of chemicals currently added and the number of chemicals expected to automatically design the plate. Once all factors are satisfied, the checklist turns fully green. Chemicals can be added manually via the form at the bottom of the page, and each reagent should be associated with a descriptive name, molar mass, desired reaction molarity, overhead or amount to prepare multiplier, SMILES, and reagent/factor type. The "screen?" checkbox indicates whether the compound should count towards the automatic plate distribution. This option is given should the user wish to automatically generate parts of the reaction array and manually adjust other portions in the **Grid** stage. Chemicals can be automatically added via a small example library of common reagents provided by clicking the "Add From Database" button. Here a searchable database of chemicals associated with a molecular weight, SMILES, a color, and descriptive name is shown. Colors can be switched by clicking on the color box associated with a chemical. Checking the boxes and assigning a reaction molarity and factor type then clicking "Add" will add the selected chemicals to the experimental reagent list. Again, chemicals can be added to this stage via CSV template with the following headers: [atp, chemicalName, chemtype, density, factor, molarMass, molarity, order, smiles], where atp (amount to prepare) is the overhead multiplier, chemtype is the factor type, and order is the anticipated order of addition into the reaction well. It is necessary for chemtype to be one of the factor types defined in the protocol (e.g., Nucleophile, Catalyst1, etc.). Examples and templates for this file can be found in the 'chemicals_input' folder of the provided GitHub repository. Should screening factors be defined and met, clicking "Run" takes the user to the **Grid** stage and automatically populates the experimental design. Otherwise, clicking the forward arrow will proceed to the **Grid** stage.

The **Grid** stage is an interactive wellplate where reagents can be manually added or removed from one or multiple wells. Clicking on a well shows detailed information about reagents added to that well and dragging over or clicking multiple wells allows for bulk edits to be made to the design. Stock solution recipes are shown in the table on the left, and true weights of reagents can be added to recalculate solvent volumes to achieve the correct stock molarity. Hit record weights to save the true weighted masses of the reagents. The download buttons provide a variety of CSV outputs to integrate with analytical or robotic instrumentation. Typically, the user will download the 'Wellplate recipe' and send this file to a printer to set up the experiment.

Once the experiment is complete and analysis data has been collected, the **Analysis** stage can visualize a series of outputs. Here, a CSV file is required with the following headers: [Sample Name, product_smiles, product_yield, product_name] where Sample Name is the well label (e.g., A1, A2, D6, etc.). Optionally, the headers [side_product1_smiles, side_product1_yield, side_product1_name, side_product2_smiles, side_product2_yield, side_product2_name] are supported for full report display. Again, examples and templates for this file can be found in the 'analysis_input' folder of the provided GitHub repository. Additional columns can be added for any analytical method. Various outputs can be displayed three at a time by choosing the headers for the heatmaps. The heatmaps are interactive, and clicking a cell displays the inputs, outputs, output value, and molecular structure of the product in the well.

Finally, the **Report** stage provides a series of outputs displaying information regarding the performed experiment. Full output requires information regarding side product 1 and side product 2, but minimally only main product information is the 'Download Results CSV' button.

c. Provided Examples.

i. 24-well esterification reaction array (Figure 2a)

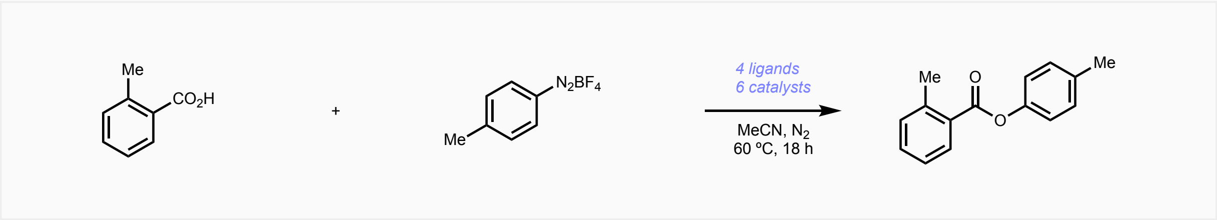

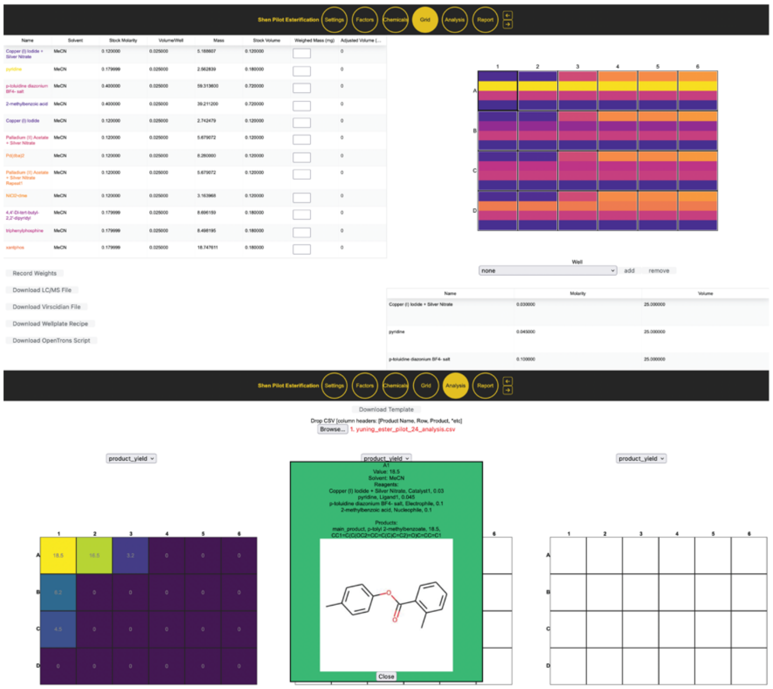

Supplementary Figure 2. Grid and Analysis stages of phactor™ for the 24-well esterification reaction array shown in Figure 2a. Four ligands were screened against six catalysts. Experimental details can be found in supplementary ref. 1.

ii. 24-well indolization reaction array (Figure 2b)

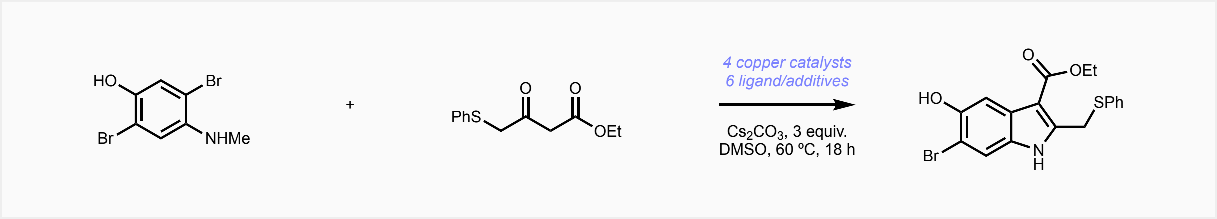

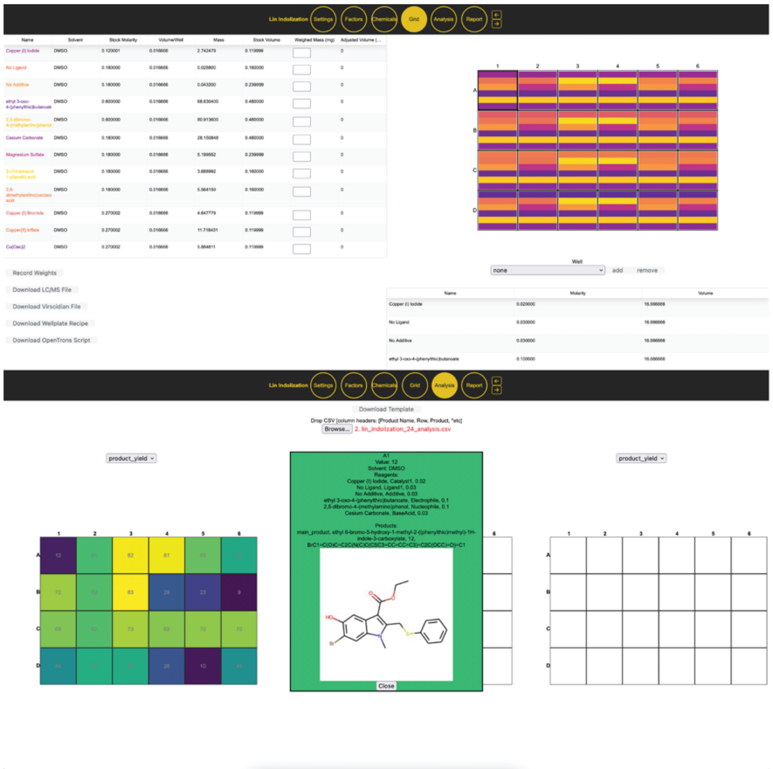

Supplementary Figure 3. Grid and Analysis stages of phactor™ for the 24-well indolization reaction array shown in Figure 2b. Experimental details can be found in supplementary ref. 2.

iii. 24-well amide pilot reaction array (Figure 3a)

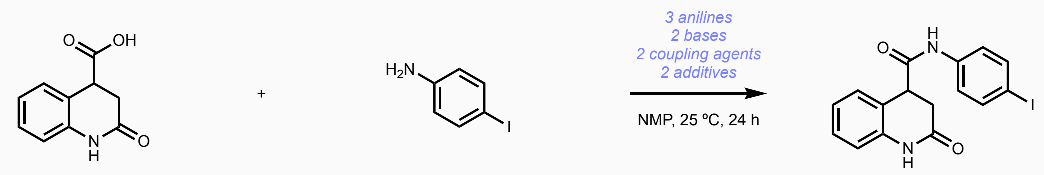

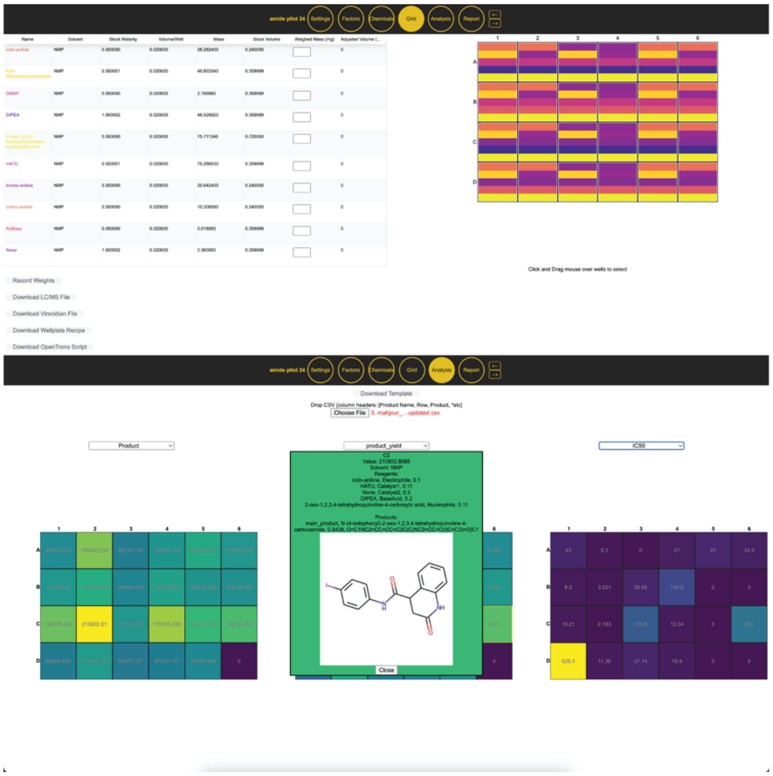

Supplementary Figure 4. Grid and Analysis stages of phactor™ for the 24-well amide coupling reaction array shown in Figure 3a. Experimental details can be found in §II.d.i.

iv. 96-well amine acid *sp*^{3}-*sp*^{3} C–C coupling (Figure 3d)

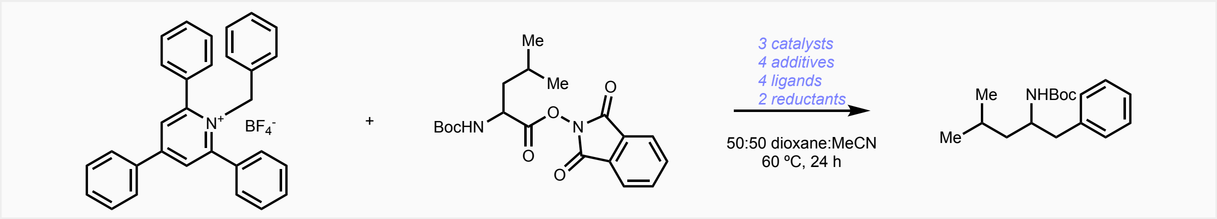

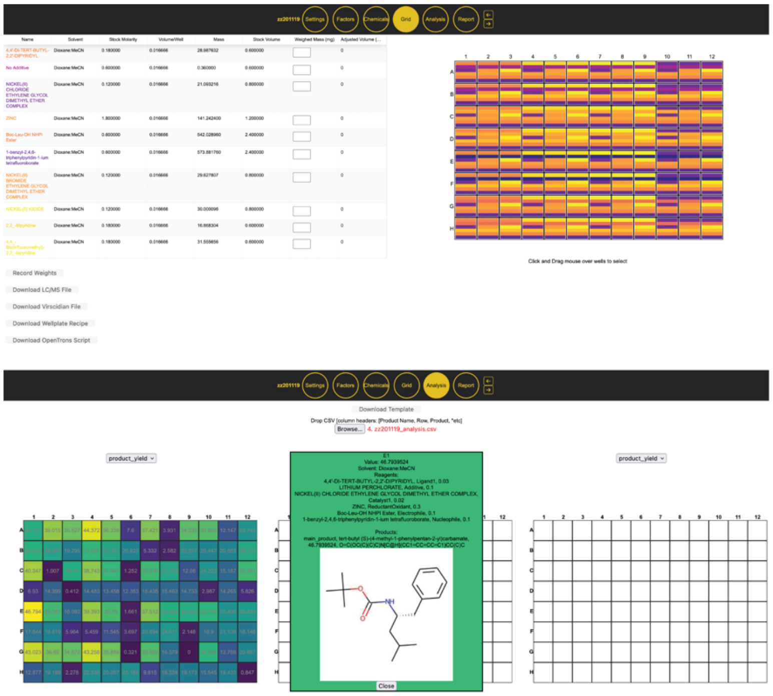

Supplementary Figure 5. Grid and Analysis stages of phactor™ for the 96-well coupling reaction array shown in Figure 3d. Experimental details can be found in supplementary ref. 3.

v. 96-well amine acid sp^3-sp^3 C–C coupling (Figure 3e)

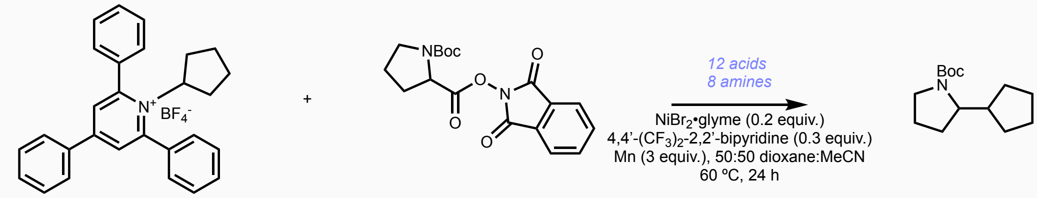

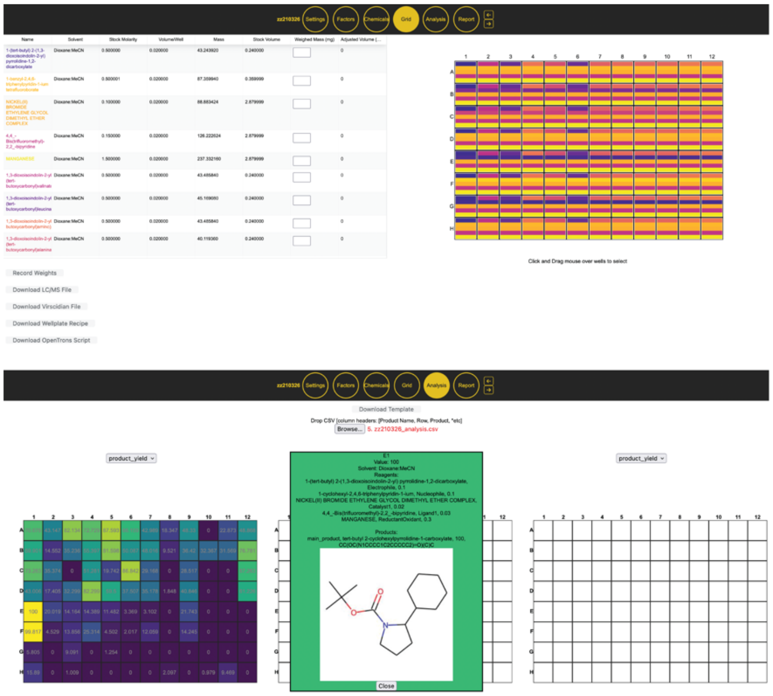

Supplementary Figure 6. Grid and Analysis stages of phactor™ for the 96-well coupling reaction array shown in Figure 3e. Experimental details can be found in supplementary ref. 3.

vi. 96-well amine acid sp^3-sp^2 C-C coupling (Figure 3f)

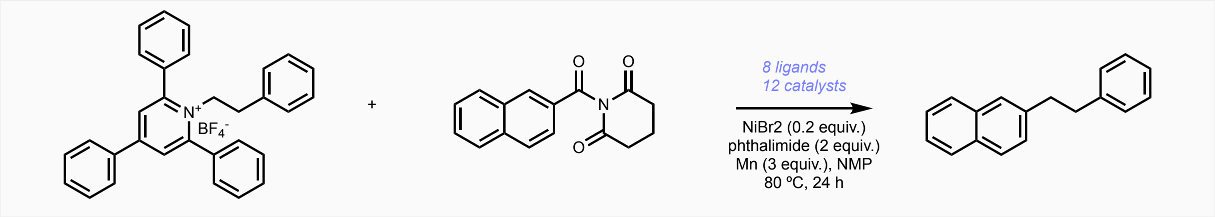

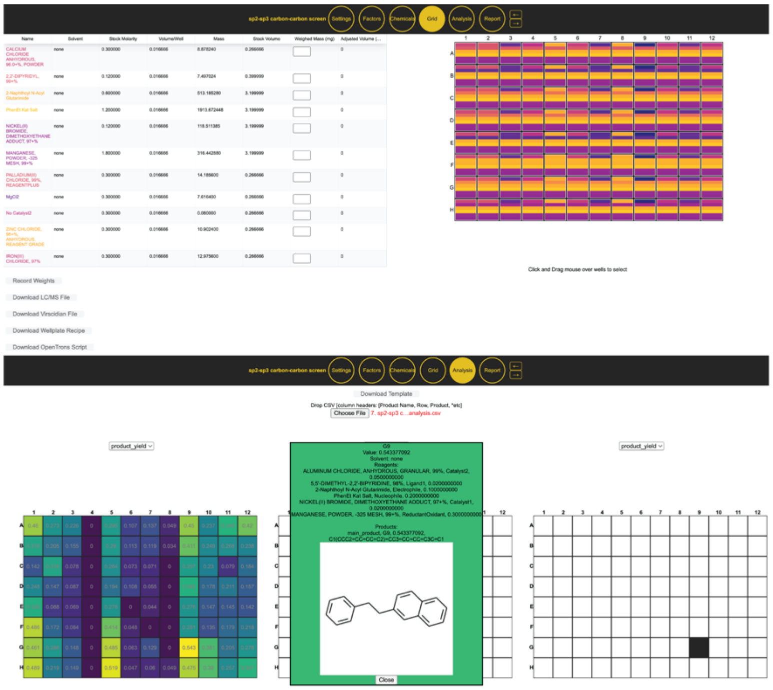

Supplementary Figure 7. Grid and Analysis stages of phactor™ for the 96-well coupling reaction array shown in Figure 3f. Experimental details can be found in §II.c.v.

d. Database.

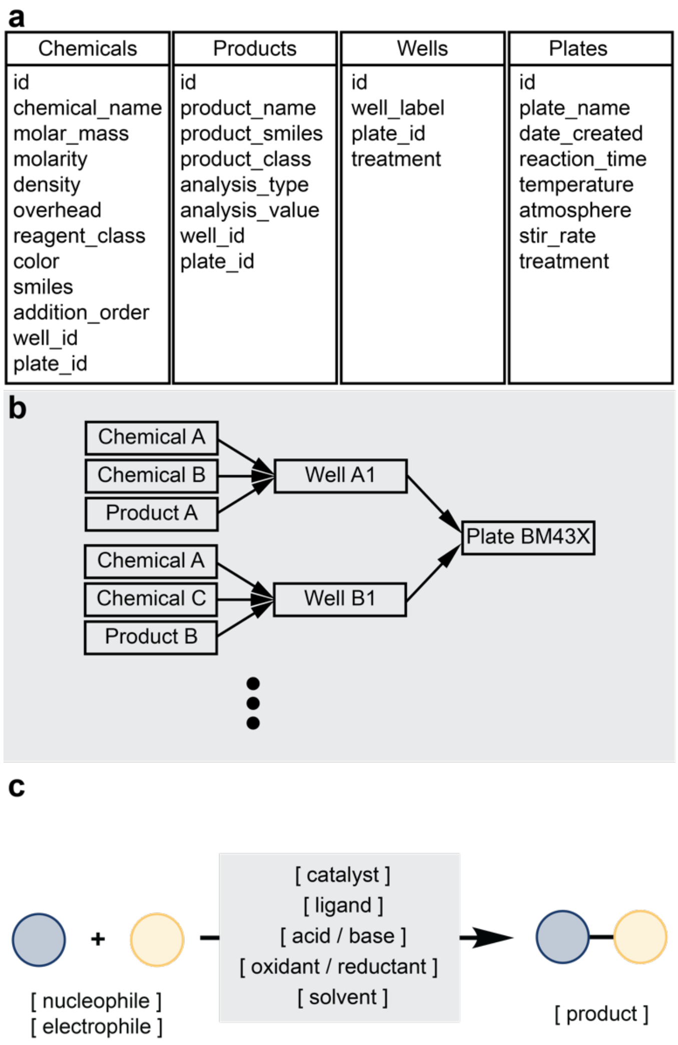

Supplementary Figure 8. a, SQL Database template for storage of HTE data in phactor™. SQL is a well understood implementation of a database that works well for reaction data. General information enabling replicability is stored, and the database can be queried at different levels, such as by plate, reagent, product, etc. **b,** Many-to-one SQL relational database formulating the data structure of HTE data. This framework allows for a variable number of reagents, products, and analytical methods to be associated with each well, and a variable number of wells to be associated with each plate. **c,** Discrete reaction layout incorporated into phactor™. Classifying the chemical species in each reaction distills reaction logic into the data framework.

e. API Connectivity.

i. Calculating Analytical Trends With phactor™'s API

a

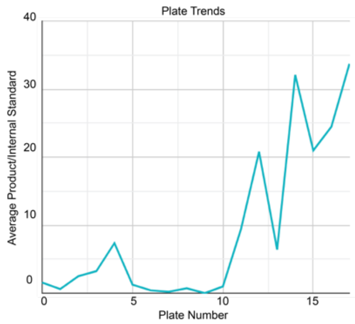

Plate Number	Size	Average Product/ Internal Standard
1	96	1.572562941395833
2	96	0.6048567708333333
3	24	2.5
4	96	3.2379891065833335
5	24	7.352901532416667
6	96	1.25
7	24	0.4166666666666667
8	24	0.20833333333333334
9	24	0.717729221875
10	96	0
11	96	0.9934375000000002
12	96	9.416092813250001
13	96	20.795771999302087
14	96	6.405667362291666

b

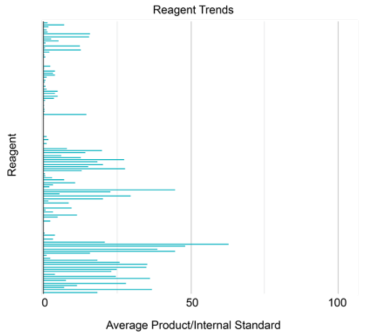

Reagent Name	SMILES	Average Product/ Internal Standard
1-(tert-butyl) 2-(1,3-dio…	CC(C)(C)OC(=O)N1C…	62.8977997568125
1-(4-methylbenzyl)-2,4,…	Cc1ccc(C[n+]2c(-c3ccc…	48.421353744375004
5,5_-Bis(trifluoromethyl…	FC(F)(F)c1ccc(-c2ccc(…	44.7589523805
1-(2,4-dichlorobenzyl)-…	Clc1ccc(C[n+]2c(-c3cc…	44.67494271091667
2,4,6-triphenyl-1-(4-(trif…	FC(F)(F)c1ccc(C[n+]2c…	38.739852451249995
1,3-dioxoisoindolin-2-yl…	CCCCC(NC(=O)OC(C)…	36.900855558124995
1,3-dioxoisoindolin-2-yl…	COc1ccc2c(c1)c(CC(=…	36.339714936250004
1,3-dioxoisoindolin-2-yl…	CCCC(NC(=O)OC(C)(…	35.289734424
1,3-dioxoisoindolin-2-yl…	CC(NC(=O)OC(C)(C)C…	35.0553893135625
2,2_-Bipyridine-5,5_-di…	O=C(O)c1ccc(-c2ccc(C…	29.86714285875
1,3-dioxoisoindolin-2-yl…	CC(C)Cc1ccc(C(C)C(=…	28.082070125
4,4_-Bis(trifluoromethyl…	FC(F)(F)c1ccnc(-c2cc(…	27.949230368809914
LITHIUM CHLORIDE	[Cl-].[Li+]	27.550950058479163
1,3-dioxoisoindolin-2-yl…	CC(C)CC(NC(=O)OC(…	26.053590453125

c

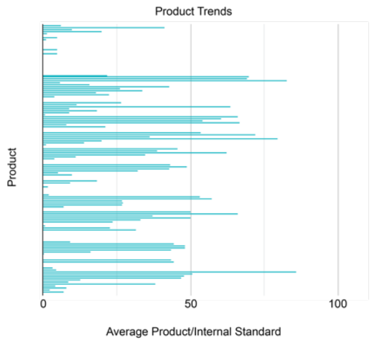

Product Name	SMILES	Average Product/ Internal Standard
tert-butyl (1-phenylhex…	CCCCC(Cc1ccccc1)N…	85.76749927
tert-butyl 2-cyclopentyl…	CC(C)(C)OC(=O)N1C…	82.685471275
tert-butyl (1-(2,4-dichlor…	CC(Cc1ccc(Cl)cc1Cl)N…	79.75
tert-butyl (1-(p-tolyl)pro…	Cc1ccc(CC(C)NC(=O)…	72.1967525
tert-butyl 2-(4-methylbe…	Cc1ccc(CC2CCCN2C(…	70.01573676000001
tert-butyl 2-(2,4-dichlor…	CC(C)(C)OC(=O)N1C…	69.205313855
tert-butyl (1-(2,4-dichlor…	CCCC(Cc1ccc(Cl)cc1C…	66.63717843500001
tert-butyl (1-phenylpent…	CCCC(Cc1ccccc1)NC(…	66.216511395
(4-chlorophenyl)(5-met…	COc1ccc2c(c1)c(CCc1…	66.18350225
tert-butyl (1-(2,4-dichlor…	CC(C)CC(Cc1ccc(Cl)c…	63.67012596
tert-butyl (4-(methylthio…	CSCCC(Cc1ccc(C(F)(…	62.51417084
tert-butyl (1-(p-tolyl)pen…	CCCC(Cc1ccc(C)cc1)…	60.55090499000001
1,2-dimethoxy-4-(4-met…	COc1ccc(CCc2ccc(C)c…	57.426702719999994
2-methoxy-6-(1-(4-(trifl…	COc1ccc2cc(C(C)Cc3c…	54.49115325

Supplementary Figure 9. Analytical trends can be rapidly analyzed with phactor™'s API. **a,** Trends of plate results over entirety of campaign. **b,** Trends of reagent results with respect to desired product yield across entirety of campaign. **c,** Trends of product results across entirety of campaign.

ii. UV difference heatmap

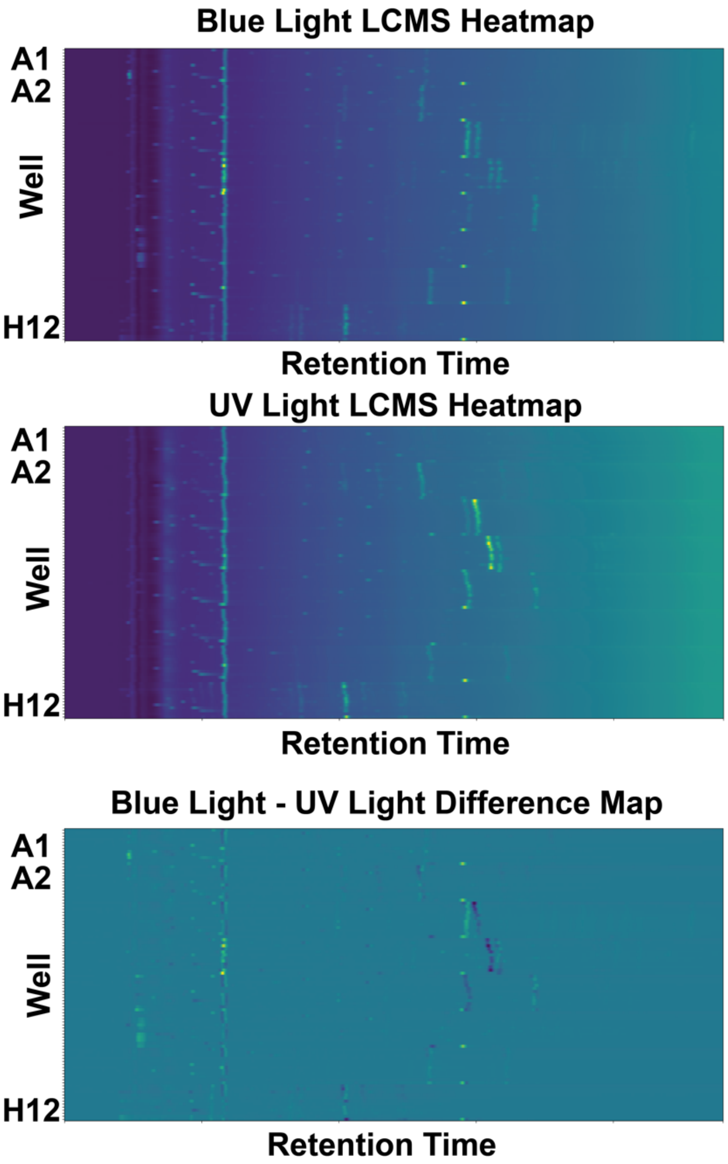

Supplementary Figure 10. LCMS Heatmaps generated from raw UPLC files for reaction arrays run under light irradiation of different colors. The difference between the blue light 96-well plate output is taken from the UV light 96-well plate output to create the difference heatmap, where yellow spots indicate higher selectivity of a product when the reaction is run in blue light and purple spots indicate high selectivity of a product in UV light.

iii. Flask API Endpoint

```
@phactor3.app.route('/api/v1/experiments/get_names/', methods=["GET"])
def get_experiments():
    """Get all screens in the database, to allow user to load from list."""
    context = {}
    context["result"] = get_experiment_names_db()
    return flask.jsonify(**context)
```

Supplementary Figure 11. Example of basic GET API endpoint exposing phactor™'s database to HTTP.

iv. React Button Example

```python
def download_opentrons():
    """Download opentrons recipes csv file."""
    context = {}
    recipe = get_bulk_info_db()
    size = session["size"]
    file = BytesIO()
    file.write(b"from opentrons import protocol_api\n")
    file.write(b"metadata = {'apiLevel': '2.0'}\n")
    file.write(b"def run(protocol: protocol_api.ProtocolContext):\n")
    file.write(b"\t#USER INPUT VALUES\n")
    file.write(b"\ttiprack_1_name = 'opentrons_96_tiprack_300ul'\n")
    file.write(b"\ttiprack_1_location = 1\n")
    file.write(b"\ttip_name = 'p300_single'\n")
    file.write(b"\ttip_head_side = 'left'\n")
    file.write(b"\tsource_plate_name = 'source_plate_holder'\n")
    file.write(b"\tsource_plate_location_1 = 4\n")
    file.write(b"\tsource_plate_location_2 = 5\n")
    file.write(b"\tdestination_plate_name = '24 or 96 plate name'\n")
    file.write(b"\tdestination_plate_location = 6\n")
    file.write(b"\ttiprack_1 = protocol.load_labware(tiprack_1_name, tiprack_1_location)\n")
    file.write(b"\tp300 = protocol.load_instrument(tip_name, tip_head_side, tip_racks=[tiprack_1])\n")
    file.write(b"\tp300.well_bottom_clearance.dispense = 20\n")
    file.write(b"\tp300.well_bottom_clearance.aspirate = 5\n")
    file.write(b"\tp300.flow_rate.aspirate = 75\n")
    file.write(b"\tp300.flow_rate.dispense = 200\n")
    file.write(b"\tair_gap = 10\n")
    file.write(b"\t#END USER INPUT VALUES\n")
    file.write(b"\tsource_plate_1 = protocol.load_labware(source_plate_name, source_plate_location_1)\n")
    file.write(b"\tsource_plate_2 = protocol.load_labware(source_plate_name, source_plate_location_2)\n")
    file.write(b"\tdestination_plate = protocol.load_labware(destination_plate_name, destination_plate_location)\n")
    source_plates = ["source_plate_1", "source_plate_2"]
    if "weighs" in session:
        weighs = session["weighs"]
    else:
        weighs = [0 for i in range(session["size"])]
    for i,k in enumerate(recipe):
        if weighs[i] != 0:
            dose = truncate(float(weighs[i])/(float(k["molarMass"]) * float(k["stockM"]))*1000, 1)
        else:
            dose = truncate(k['mls'][0]*1000, 1)

        file.write(f"\tp300.distribute({dose}, {source_plates[k['source_plate']]}['{k['source_well']}'], \
            [dest.wells_by_name()[well] for well in {k['locs']}], new_tip = 'once', air_gap = air_gap])\n".encode())
    file.seek(0)
    return flask.send_file(file, mimetype='text/py')
```

Supplementary Figure 12. Example JavaScript/React code for a button component that calls phactor™'s API.

v. **OpenTrons OT-2 Conversion API Example**

```javascript
import React from 'react';
import '../../../static/buttons.css';

export default function OpenTronsButton() {

  function downloadOpenTrons() {
    fetch('/api/v1/grid/opentrons_download/', { credentials: 'same-origin' })
      .then((response) => {
        if (!response.ok) throw Error(response.statusText);
        response.blob().then((blob) => {
          const url = window.URL.createObjectURL(blob);
          const a = document.createElement('a');
          a.href = url;
          a.download = screenName+'_opentrons.py';
          a.click();
        });
      })
      .then((data) => {
        console.log(data); // eslint-disable-line no-console
      })
      .catch((error) => console.log(error)); // eslint-disable-line no-console
  }

  return (
    <div>
      <button className="opentronsButton" type="submit" onClick={() => downloadOpenTrons()}> Download OpenTrons Script </button>
      {' '}
      <br />
    </div>
  );
}
```

Supplementary Figure 13. 45 lines of code needed to interface phactor™ with the OpenTrons OT-2 robot.

f. Conversion Scripts

i. Open Reaction Database (ORD) Conversion Script

```python
from ord_schema.proto import dataset_pb2, reaction_pb2
from ord_schema import message_helpers
from rdkit import Chem
from rdkit import RDLogger
RDLogger.DisableLog('rdApp.*')
import pandas as pd

data = pd.read_csv("5. zz210326_final_results.csv")

component_types = ["Electrophile", "Nucleophile", "Catalyst1", "Ligand1", "Ligand2", \
                   "Catalyst2", "BaseAcid", "ReductantOxidant", "Additive", "Solvent1", "Solvent2", "Product"]
component_smiles = [k+"_SMILES" for k in component_types]
component_charges = [k+" charge" for k in component_types]

convert_data = {k:[] for k in component_types}
for k in component_smiles:
    convert_data[k] = []
for k in component_charges:
    convert_data[k] = []
convert_data["Pd/IS"] = []
convert_data["Plate Position"] = []
convert_data["Reaction_type"] = []

for i,k in data.iterrows():
    for j in component_types:
        convert_data[j].append(k[j])
        if j == "Solvent1" or j == "Solvent2":
            sm = "None"
        else:
            sm = k[j+" SMILES"]
        if sm == None or sm == "None":
            convert_data[j+"_SMILES"].append("None")
        else:
            sm = Chem.MolFromSmiles(sm)
            if sm != None:
                sm = Chem.MolToSmiles(sm)
            else:
                sm = "None"
            convert_data[j+"_SMILES"].append(sm)
        if j == "Solvent1" or j == "Solvent2" or j == "Product":
            convert_data[j+" charge"].append("None")
        else:
            convert_data[j+" charge"].append(f"{k[j+ ' Conc (M)']}")
    convert_data["Pd/IS"].append(k["Output Value"])
    convert_data["Plate Position"].append(k["Row"] + str(k["Column"]))
    convert_data["Reaction_type"].append("sp3-sp3 deaminative-decarboxylative C-C coupling")
out_data = pd.DataFrame(convert_data)

reaction_dataset = dataset_pb2.Dataset()
for i,k in out_data.iterrows():
    reaction = reaction_pb2.Reaction()
    for rxt in ["Electrophile", "Nucleophile", "Catalyst1", "Ligand1", "Ligand2", "Catalyst2", "BaseAcid", "ReductantOxidant", "Additive"]:
        if k[rxt] == "None":
            continue
        solute = reaction.inputs[rxt].components.add()
        solute.CopyFrom(
            message_helpers.build_compound(
                name=k[rxt],
                smiles=k[rxt+"_SMILES"],
                role="reactant",
                amount=f"{float(k[rxt+' charge'])*.1} mmol",
                prep=None,
                is_limiting=False,
                prep_details=None,
            )
        )
    solvent = reaction.inputs["Solvent1"].components.add()
    solvent.CopyFrom(
        message_helpers.build_compound(
            name=k["Solvent1"],
            smiles="placeholder",
            role="solvent",
            amount="100 uL",
            prep=None,
            is_limiting=False,
            prep_details=None,
        )
    )
    solvent.amount.volume_includes_solutes = True

    outcome = reaction.outcomes.add()
    prod_2a = outcome.products.add(is_desired_product=True)
    prod_2a.identifiers.add(type="SMILES", value=k["Product_SMILES"])
    prod_2a.identifiers.add(type="NAME", value=k["Product"])
    prod_2a.reaction_role = reaction_pb2.ReactionRole.PRODUCT
    prod_2a.measurements.add(type="YIELD", analysis_key="UPLC-MS Integration", percentage=dict(value=k["Pd/IS"], precision=5), uses_internal_standard=True
                            )
dataset = dataset_pb2.Dataset(
    name="sp3-sp3 deaminative-decarboxylative C-C coupling zz210326",
    description="DOI: 10.1002/anie.202112454",
    reactions=reactions,
)
message_helpers.write_message(dataset, 'ord-zz210326.pbtxt')
```

Supplementary Figure 14. Interfacing code between the output of phactor™ and ORD.

ii. Chemical Description Language (XDL) Conversion Script

```python
import xml.etree.cElementTree as ET
import pandas as pd

data = pd.read_csv("5. zz210326_final_results.csv")
data_stocks = pd.read_csv("5. zz210326_recipe.csv")

root = ET.Element("XDL")
synthesis = ET.SubElement(root, "Synthesis")
reagents = ET.SubElement(synthesis, "Reagents")
component_types = ["Electrophile", "Nucleophile", "Catalyst1", "Ligand1", \
                   "Ligand2", "Catalyst2", "BaseAcid", "ReductantOxidant", "Additive"]

for i,k in data_stocks.iterrows():
    ET.SubElement(reagents, "Reagent",
                  name=k["name"],
                  id=k["name"],
                  role="reagent"
                 )

for i2, k2 in data.iterrows():
    reagents = ET.SubElement(synthesis, "Procedure", name=k2["Row"]+str(k2["Column"]))
    for i3 in component_types:
        if k2[i3] == None or k2[i3] == "None":
            continue
        reagent2 = ET.SubElement(reagents, "Add",
                                 name=k2[i3],
                                 volume="200 uL")

tree = ET.ElementTree(root)
tree.write("filename.xml")
```

Supplementary Figure 15. Interfacing code between the output of phactor™ and XDL.

iii. Experimental Design via Bayesian Optimization+ (EDBO+) Conversion Script

```python
from rdkit import Chem
from rdkit import RDLogger
RDLogger.DisableLog('rdApp.*')
import pandas as pd

data = pd.read_csv("5. zz210326_final_results.csv")
component_types = ["Electrophile", "Nucleophile", "Catalyst1", "Ligand1", "Ligand2", "Catalyst2", \
                   "BaseAcid", "ReductantOxidant", "Additive", "Solvent1", "Solvent2", "Output Value"]
component_amounts = {k:[] for k in component_types}
for i,k in data.iterrows():
    for j in component_types:
        component_amounts[j] = k[j]
data.to_excel("zz20326_EDBO+_input.xlsx")
```

Supplementary Figure 16. Interfacing code between the output of phactor™ and EDBO+.

Supplementary Methods

II. **Experimental**

a. **General Information.** Code for phactor™ was written in Python (version 3.9.10) and ReactJS (version 18.2.0) with minimal dependencies. Python dependencies were limited to Flask (version 2.0.2), Numpy (version 1.22.2), Pandas (1.4.1), Matplotlib (version 3.5.1), and RDKit (version 2021.09.4), all installed via pip (version 22.0.3). JavaScript dependencies were limited to ReactJS (version 18.2.0) for the underlying user interface infrastructure, react-csv-reader (version 3.3.0) and react-table-6. API endpoints were written in Flask and exposed via HTTPS.

All chemical reactions were conducted in oven- or flame-dried glassware and set up in a fumehood exposed to air. All solvents and reagents were purchased from Sigma Aldrich, Alfa Aesar, Oakwood Chemical, or TCI Chemical and were used as received. Glass 2-dram vials (ChemGlass #CG-4912-02) were used as reaction vessels, fitted with a screw-cap with a Teflon-coated silicone septa (CG-4910-02), and magnetic stir bars (Fisher Scientific #14-513-93 or #14-513-65). Proton nuclear magnetic resonance spectra (1H NMR) were recorded on a Varian MR-500 MHz spectrometer and chemical shifts are reported in parts per million (ppm) using the solvent residual peak as an internal standard (DMSO6 at 2.54 ppm). Reaction analysis was typically performed by thin-layer chromatography on silica gel, or using a Waters I-class ACQUITY UPLC-MS (Waters Corporation, Milford, MA, USA) equipped with in-line photodiode array detector (PDA) and QDa mass detector (ESI positive ionization mode). 0.1 µL sample injections were taken from acetonitrile solutions of reaction mixtures or products (~1 mg/mL). A partial loop injection mode was used with the needle placement at 1.0 mm from bottom of the wells and a 0.2 µL air gap at pre-aspiration and post-aspiration. Column used: Waters Cortecs UPLC C18+ column, 2.1mm x 50 mm with (Waters #186007114) with Waters Cortecs UPLC C18+ VanGuard Pre-column 2.1mm x 5 mm (Waters #186007125), Mobile Phase A: 0.1% formic acid in Optima LC/MS-grade water, Mobile Phase B: 0.1% formic acid in Optima LC/MS-grade MeCN. Flow rate: 0.8 mL/min. Column temperature: 45 ºC. The PDA sampling rate was 20 points/sec. The QDa detector monitored m/z 150-750 with a scan time of 0.06 seconds and a cone voltage of 30 V. The PDA detector range was between 210 nm – 400 nm with a resolution of 1.2 nm. A 2-minute method was used. The method gradients are below: 0 min: 0.8 mL/min, 95% 0.1% formic acid in water/5% 0.1% formic acid in acetonitrile; 1.5 min : 0.8 mL/min, 0.1% 0.1% formic acid in water/99.9% 0.1% formic acid in acetonitrile; 1.91 min : 0.8 mL/min, 95% 0.1% formic acid in water/5% 0.1% formic acid in acetonitrile. Thin Layer Chromatography was performed on 25 µm TLC Silica gel 60 F254 glass plates purchased from Fisher Scientific (part number: S07876). Visualization was performed using ultraviolet light (254 nm).

b. **Inhibition Assay General Information.**

Chemicals and solvents: HEPES was purchased from Fisher BioReagents, DTT was purchased from research product international, EDTA was purchased from Acros Organics, DMSO was purchased from Fisher scientific, LC-MS solvents were of Optima® LC-MS grade supplied by Fisher Chemical and filtered/degassed through the 0.45 µm polytetrafluoroethylene (PTFE) membrane prior to use. M^{Pro} enzyme protein was supplied by Center for Structural Biology and the substrate peptide was supplied by the Proteomics and Peptide Synthesis core at the University of Michigan.

Instruments: M^{Pro} enzyme, peptide substrate, and formic acid solutions were all dispensed using a Multidrop Combi reagent dispenser (Thermo Scientific), the plates were centrifuged (1000 x g, 60s, Beckman Coulter Plate Spinner Centrifuge, Beckman Coulter). The 384 plates were sealed with Agilent pierceable aluminum seal using Agilent Plateloc Thermal microplate sealer with the following parameters (seal time: 1.2 s; seal temperature 170 ºC; air pressure 81 psi). M^{Pro} activity was assayed using a RapidFire (RF) 365 high-throughput sampling robot (Agilent) connected to an Agilent 6545 ESI-Q-TOF mass spectrometer (acquired by Natural Products Discovery Core at the University of Michigan). A solid-phase extraction (SPE) C18-cartridge was utilized with the following method: 0.1% (v/v) aqueous formic acid (4 s, 1.25 mL/min) and with aqueous 85% (v/v) acetonitrile containing 0.1% (v/v) formic acid (8 s, 0.8 mL/min). The cartridge was re-equilibrated with 0.1% (v/v) aqueous formic acid (0.7 s, 1.25 mL/min). Each sample aspiration step was followed by a blank injection before the next protein sample injection. The mass spectrometer was operated in the positive ion ionization mode with following operating parameters for all assays: capillary voltage (3500 V), nozzle voltage (500 V), fragmentor voltage (140 V), drying gas temperature (325 °C), gas flow (10 L/min), sheath gas temperature (300 °C), sheath gas flow (10 L/min).

c. **Selected Screening Examples**

i. **Furan Indolization Reaction Array Procedure**

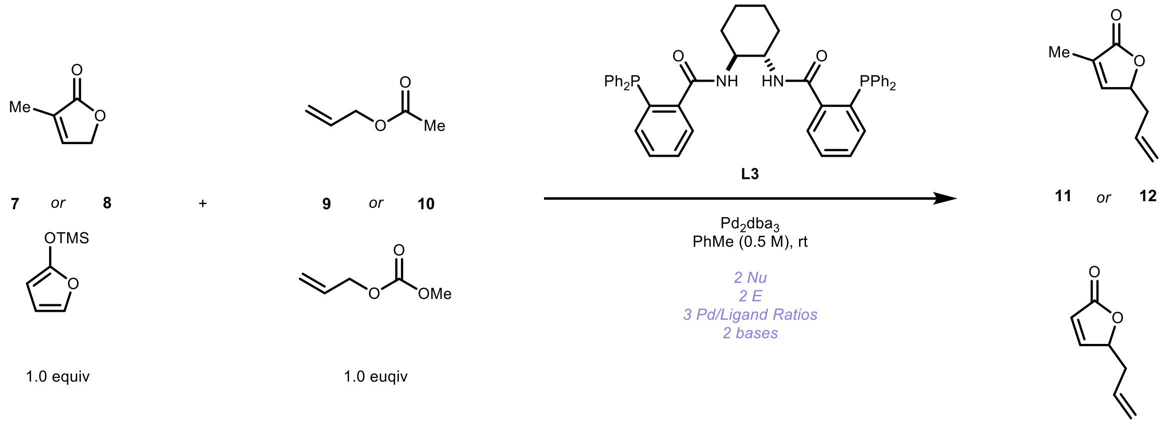

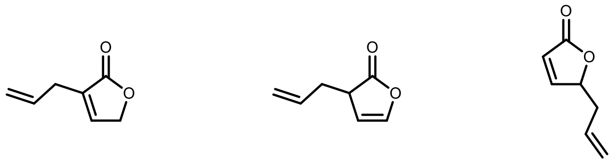

α1 coupling α2 coupling γ coupling

Two nucleophiles **7** or **8** (1.0 equiv), two electrophiles **9** or **10** (1.0 equiv), Pd_2dba_3 (5 mol%), Pd_2dba_3 (10 mol%), ligand **L3** (5 mol%), ligand **L3** (10 mol%), potassium carbonate (1.0 equiv) were prepared as stock solutions in anhydrous toluene (0.5 M) based on calculations performed by phactor™ with significant overhead. Stock solutions distributed into their respective positions on a 24 wellplate via a single channel or multichannel pipette. 20 µL of each stock solution was distributed into the reaction plate resulting in 100 µL reaction volume (0.5 M). Once all stock reagents were distributed into the reaction plate, the plate was sealed, and allowed to react for 24 hours at room temperature. Once the reactions were complete, the plate was unsealed and each reaction well was dried via N_2 blowing, followed by the addition of $CDCl_3$ (700 µL) to each reaction vial. Insoluble base and other particles were removed by filtration over a plug of Celite. The reactions were analyzed by ^{1}H NMR to determine conversion and selectivity. Multiplexed pie charts generated by phactor™ as shown in Figure 2c of the main text reveal the conditions of well D3, with a 2:1 palladium catalyst to ligand loading and no base, to generate the desired γ-regioisomer with the greatest selectivity.

ii. Mannich Reaction Array Procedure

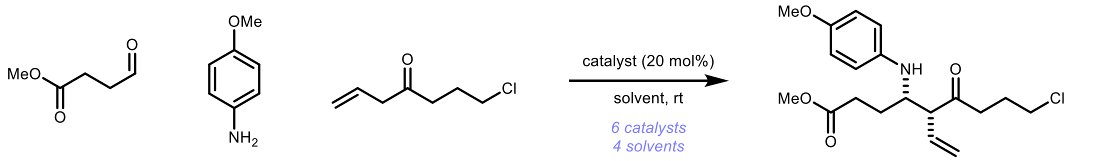

13 , 1.0 equiv **14** 1.2 equiv **15** , 2.0 equiv solvent = DMSO, THF, MeCN, PhMe **16**

catalyst:

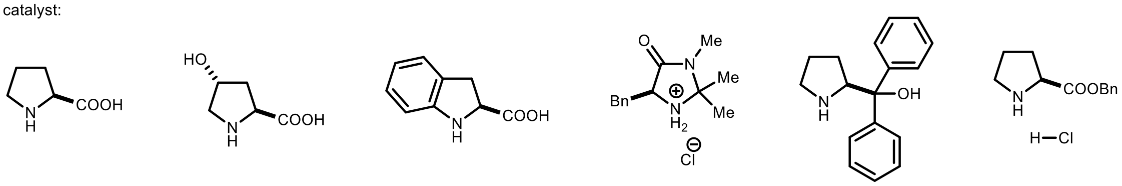

C1 **C2** **C3** **C4** **C5** **C6**

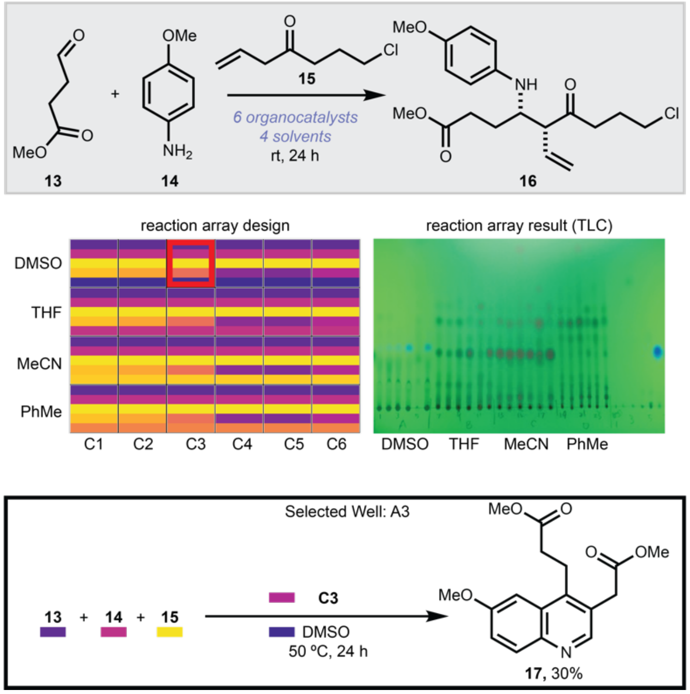

Supplementary Figure 17. One of six chiral secondary amine catalysts **C1** - **C6** (see Supplementary Information) at 20 mol% loading was added to the three starting materials, and the reaction was run in one of four different solvents: DMSO, THF, acetonitrile, or toluene. After 24 hours at room temperature, the reaction array was quenched and analysed yielding a bright fluorescent spot at Rf value 0.4. Well A3 was scaled up with slight modification to confirm the fluorescent compound as undesired quinoline product **17** in 30% isolated yield (60% to limiting reagent **13**), derived from two equivalents of aldehyde **13**.

Six catalysts (20 mol%, see below), aldehyde **13** (1.0 equiv), aniline **14** (1.2 equiv), ketone **15** (2.0 equiv) were prepared as stock solutions in four separate solvents (DMSO, THF, MeCN, PhMe, 0.1 M) based on calculations performed by phactor™ with significant overhead. Stock solutions distributed into their respective positions on a 24 wellplate via a single channel or multichannel pipette. 25 µL of each stock solution was distributed into the reaction plate resulting in 100 µL reaction volume (0.1 M). Once all stock reagents were distributed into the reaction plate, the plate was sealed, and allowed to react for 24 hours at room temperature. Once the reactions were complete, the plate was unsealed, and each reaction well was spotted on a TLC plate and developed in 30% EtOAc/hexane to visualize under UV lamp as shown. An unexpected bright fluorescent spot at Rf value 0.4 was noticed and later confirmed to be a quinoline derivative (see below for details).

iii. Synthesis of 7-chlorohept-1-en-4-one (15)

Following a known procedure,[4] aluminum trichloride (0.267 g, 2.000 mmol, 0.4 equiv) was added in one portion to a solution of zinc powder (1.308 g, 20.000 mmol, 4 equiv), 4-chlorobutanenitrile (0.473 mL, 5.000 mmol, 1 equiv) and allyl bromide (0.649 mL, 7.500 mmol, 1.5 equiv) in anhydrous THF (25.0 mL) at 0 °C in an ice bath. The mixture was warmed to room temperature and stirred for 12 h before the addition of 2 M HCl (25.0 mL). The mixture was filtered through Celite and the layers were separated. The aqueous layer was extracted with EtOAc (2 × 40.0 mL). The combined organic layers were washed with brine (30.0 mL), dried over anhydrous Na_2SO_4, filtered and concentrated *in vacuo*. The crude residue was purified by flash chromatography (silica gel, eluent: 10% EtOAc/hexane) to afford **15** (353.0 mg, 48% yield) as a colorless oil.

^{1}H NMR (400 MHz, $CDCl_3$) ∂ 5.92 (ddt, *J* = 17.2, 10.2, 7.0 Hz, 1H), 5.24 – 5.10 (m, 2H), 3.57 (t, *J* = 6.3 Hz, 2H), 3.19 (dt, *J* = 7.0, 1.4 Hz, 2H), 2.65 (t, *J* = 7.0 Hz, 2H), 2.04 (p, *J* = 6.6 Hz, 2H). ^{13}C NMR (100 MHz, $CDCl_3$) ∂ 207.59, 130.45, 119.24, 48.03, 44.54, 38.95, 26.32.

The characterization data matched spectral values from literature.[5]

iv. Synthesis of methyl 3-(6-methoxy-3-(2-methoxy-2-oxoethyl)quinolin-4-yl)propanoate (17)

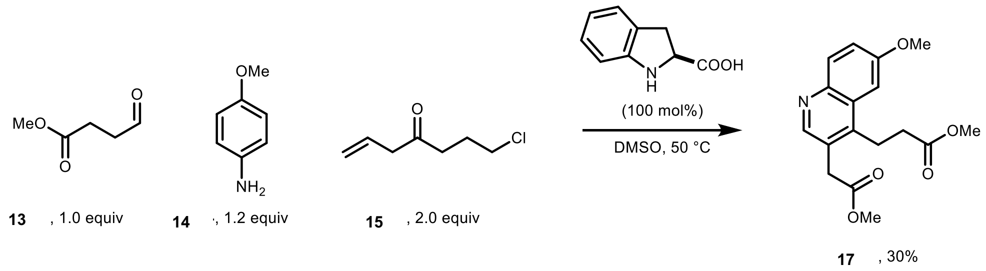

Well A3 was scaled up with slight modifications: to a mixture of 4-methoxyaniline **14** (14.8 mg, 0.120 mmol, 1.2 equiv) and (*S*)-indoline-2-carboxylic acid (16.3 mg, 0.100 mmol, 1 equiv) in DMSO (1.0 mL) was added methyl 4-oxobutanoate **13** (11.6 mg, 0.100 mmol, 1 equiv) and ketone **15** (29.3 mg, 0.200 mmol, 2 equiv) at room temperature. The mixture was stirred at 50 °C for 24 h. The mixture was diluted with water (10.0 mL) and extracted with EtOAc (3 × 5.0 mL). The combined organic layers were washed with brine (4.0 mL), dried over anhydrous Na_2SO_4, filtered and concentrated *in vacuo*. The crude residue was purified by flash chromatography (silica gel, eluent: 30% EtOAc/hexane) to afford **17** (9.6 mg, 30% yield) as a yellow oil. Proton NMR for this compound is displayed in NMR section of the Supplementary Information.

^{1}H NMR (500 MHz, $CDCl_3$) ∂ 7.87 (d, *J* = 9.2 Hz, 1H), 7.84 (s, 1H), 7.30 (dd, *J* = 9.2, 2.8 Hz, 1H), 7.00 (d, *J* = 2.8 Hz, 1H), 3.90 (s, 3H), 3.85 (s, 2H), 3.72 (s, 3H), 3.69 (s, 3H), 3.23 (t, *J* = 7.2 Hz, 2H), 2.98 (t, *J* = 7.2 Hz, 2H). ^{13}C NMR (125 MHz, $CDCl_3$) ∂ 174.26, 171.51, 157.66, 156.40, 143.25, 136.02, 130.33, 127.98, 126.58, 121.93, 104.82, 55.65, 52.44, 51.75, 38.33, 32.02, 29.76. HRMS (ESI): calculated $C_{17}H_{20}NO_5^+$ [M + H]$^+$: 318.1336, found: 318.1336.

v. *sp*^{2}–*sp*^{3} Decarboxylative Deaminative C–C Coupling Procedure

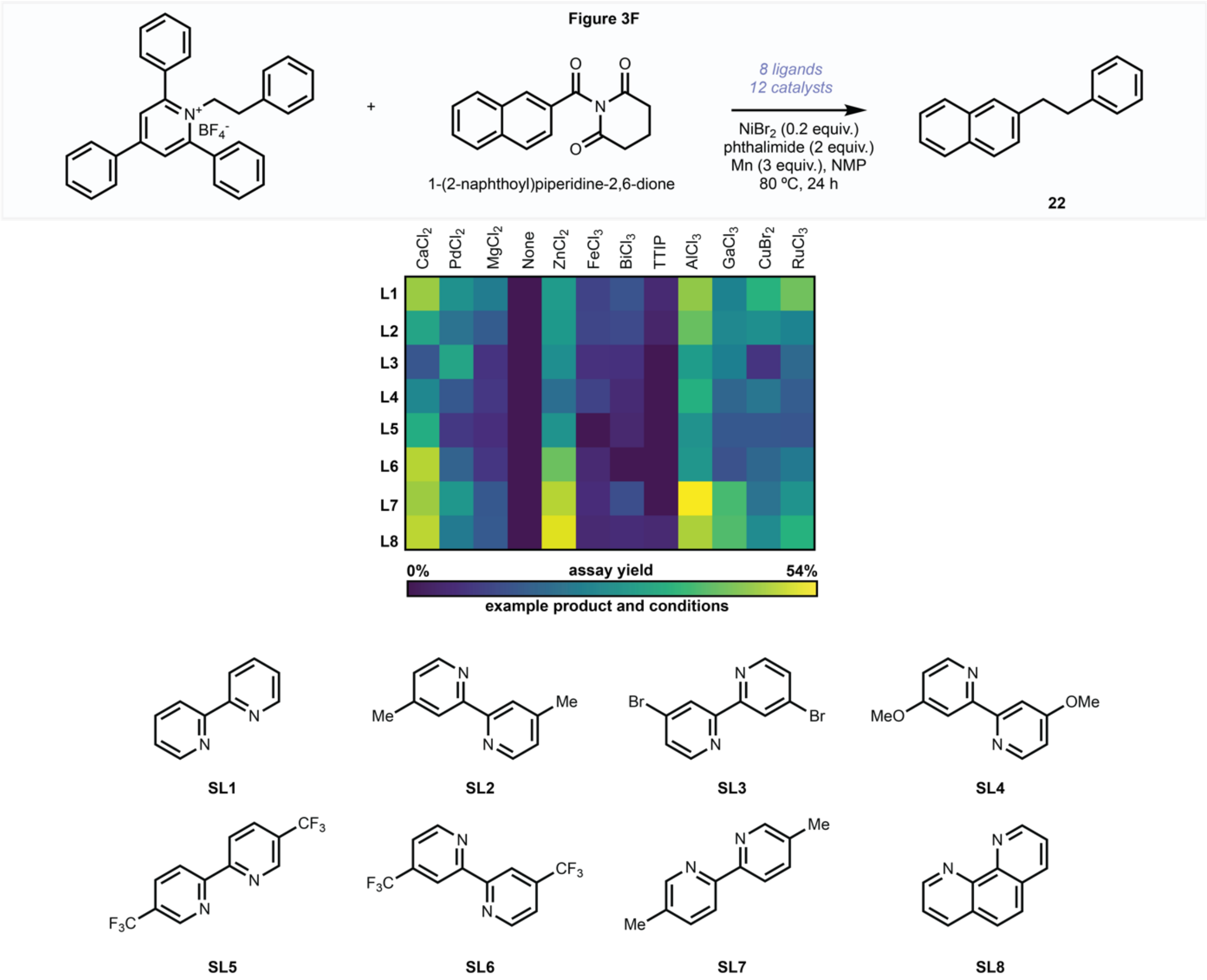

Supplementary Figure 18. Input design and results of *sp*^{2}–*sp*^{3} Decarboxylative Deaminative C–C Coupling shown in Figure 3f.

Benzyl pyridinium tetrafluoroborate salt (2.0 equiv.), 1-(2-naphthoyl)piperidine-2,6-dione (1.0 equiv), Nickel II Bromide (0.2 equiv.) , phthalamide (2.0 equiv.) , manganese (3.0 equiv.) , 8 ligands (0.2 equiv.) , and 12 catalysts (0.5 equiv.) were prepared as stock solutions in NMP (0.1 M) based on calculations performed by phactor™ with significant overhead. Stock solutions were dosed into a 96-well plate based on positions provided by phactor™. The microvial plate was sealed and moved to a ChemGlass stirring hotplate for 24 hours with stirring at 80 ºC. The reactions were quenched by opening the reaction block and exposing the reactions to air so that the reactions could be diluted to 10 mM with 9:1 DMSO:Water solution. From each reaction, an aliquot of quenched reaction mixture was added to DMSO-biphenyl solution where biphenyl served as an equimolar internal standard. The reactions were then analyzed by UPLC-MS using the analytical method described above.

vi. **ultraHTE Exploratory Amine Acid Aryl Esterification Procedure.** 48 acids (1 eq.), 8 amines (1.5 eq.) activated as their diazonium salts, lutidine (1.5 eq.), and Copper(I) tetra(acetonitrile) tetrafluoroborate (1 eq.) were prepared as stock solutions in benzonitrile based on calculations performed by phactor™ with significant overhead. Acids and diazoniums are shown in the following Supplementary Figure 20. Stock solutions were plated in a 384-well stock solution wellplate as mapped by NanoChem and distributed into their respective positions on a 1,536 wellplate with the Mosquito® robot as shown in Supplementary Figure 19. 250 µL of each stock solution was distributed into the reaction plate in addition to 200 µL from solvent wash resulting in 1.2 µL reaction volume (0.1 M). Once all stock reagents were distributed into the reaction plate, the plate was centrifuged, sealed, and allowed to react for 20 hours at room temperature. Once the reactions were complete, the plate was unsealed and each reaction well was diluted to 10 mM with 9:1 DMSO:Water solution. The mosquito® robot was used to distribute the quenched reactions into four UPLC-MS plates containing equimolar caffeine as internal standard. Product over internal standard results were visualized in phactor™ as shown in Figure 3i of the main text.

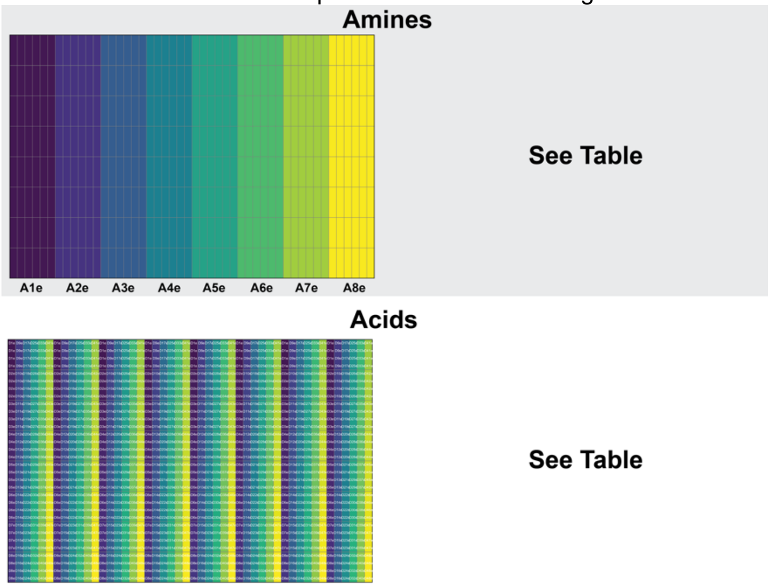

Supplementary Figure 19. Input design of the ultraHTE Exploratory Amine Acid Aryl Esterification.

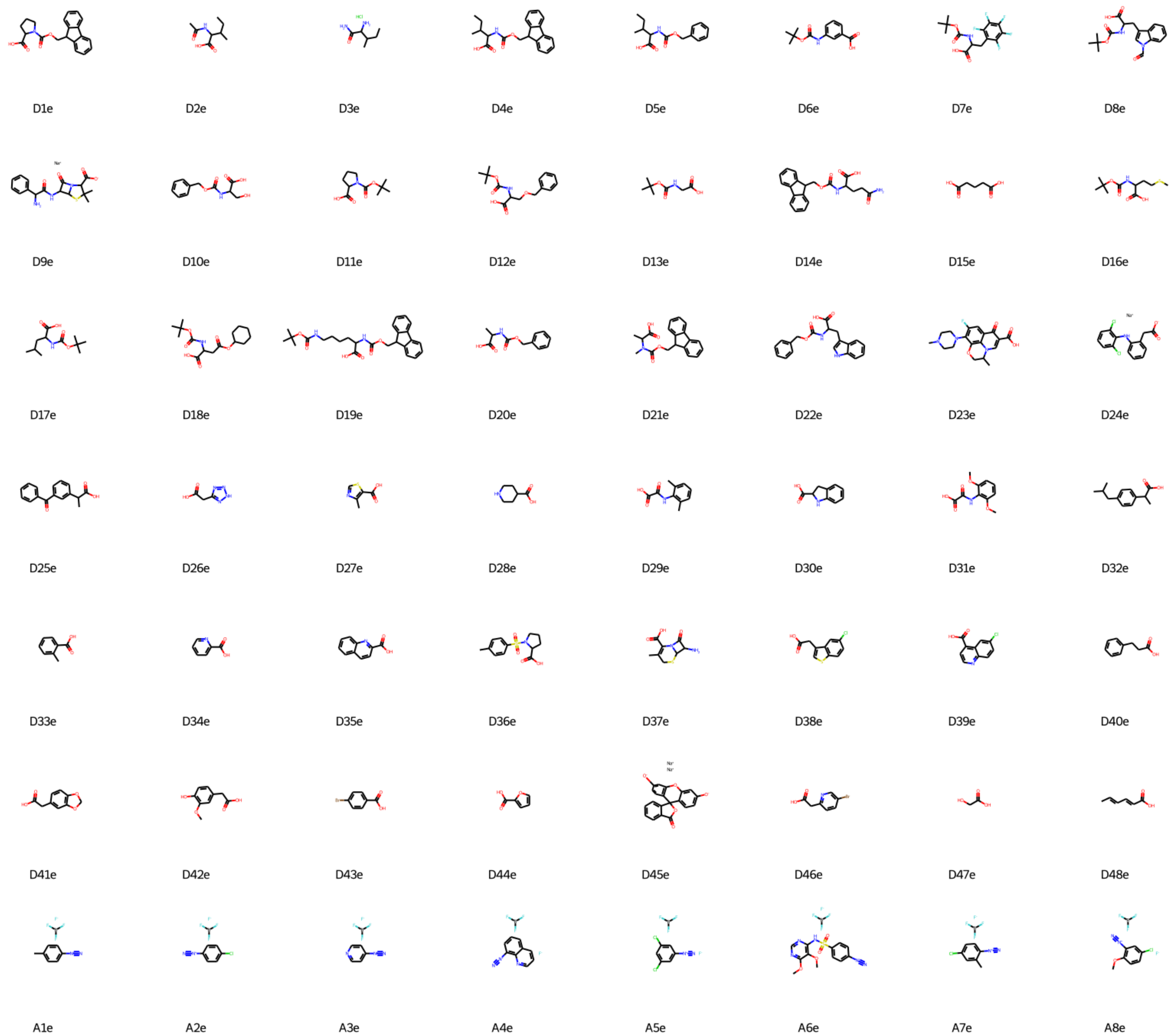

Supplementary Figure 20. Input acids and diazoniums corresponding to the design layout of Supplementary Figure 19.

d. M^{Pro} inhibitor discovery direct-to-biology with phactor™.

i. Pilot Amide Reaction Array Procedure. Stock solutions, or suspensions, were prepared as shown in Supplementary Figure 21 (anilines 0.1 M, acid 0.11 M, coupling agents 0.11 M, base 0.2 M, additive 0.01 M in 0.1 mL dry NMP). Reagents were weighed and dissolved or suspended in anhydrous degassed NMP to achieve the desired stock concentration as calculated by phactor™. Stock solutions of reagents were stirred until either a clear solution or a uniform slurry was achieved. A 24-well aluminum microvial plate (Analytical Sales & Services cat. no. 25243) was equipped with oven-dried shell vials (Analytical Sales & Services cat. no. 884001). Stock solutions were dosed to the appropriate shell vials according to the plate map shown in Supplementary Figure 21b using single channel micropipetters. A parylene-coated stir dowel (Analytical Sales & Services cat. no. 13258) was then added to each vial. The microvial plate was sealed and moved to a ChemGlass stirring hotplate for 24-hours with stirring at room temperature. The reactions were quenched by opening the reaction block and exposing the reactions to air so that the reactions could be diluted to 10 mM with 9:1 DMSO:Water solution. From each reaction, an aliquot of quenched reaction mixture was added to DMSO-biphenyl solution where biphenyl served as an equimolar internal standard. The reactions were then analyzed by UPLC-MS using the analytical method described above. Another aliquot was taken from each quenched reaction mixture and serially diluted for a biological assay to test compound IC_{50} values as described. Chemistry and biology assay results are shown in Supplementary Figure 21b-c and compared to literature values shown in Supplementary Figure 21a.

ii. M^{Pro} inhibition assay. The M^{Pro} inhibition assay was adopted and modified from the method reported by Schofield.[62] Freshly thawed M^{Pro} enzyme (1 mg/mL, 29.5 μM) was diluted to 0.3 μM in 50 mM HEPES buffer pH 7.3, 1 mM EDTA, 1 mM DTT. The substrate peptide (400 μM in DMSO) was diluted to 4 μM in the M^{Pro} buffer. An aliquot (1 or 2 μL) of each compound concentration was transferred to 384 plate. Compounds **27**, **28**, and **29**, were dispensed in a 16-point 2-fold dilution series with 100 μM as top final concentration. M^{Pro} (0.30 μM) was then

dispensed across the plate (50 µL/well) so the final concentration of DMSO is 2% or 4%. The resulting mixture was incubated for 60 minutes at room temperature. TSAVLQ/SGFRK-NH2 peptide solution (4 µM) was then added to the mixture (50 µL/well). The plate was shaken for 10 seconds, and the reactions were incubated for 10 minutes, then quenched by addition of 10% (v/v) aqueous formic acid (10 µL/well). The plates were shaken for 10 seconds after the dispensing of M^{Pro} enzyme, peptide substrate, and formic acid solutions. Ebselen (100 µM) and DMSO were used as positive and negative controls, respectively. The plates were heat sealed with pierceable aluminum seal and the reaction solutions were subjected to RF-HRMS analysis.

RapidFire integrator software (Agilent) was used to extract and integrate the m/z (+1) charge states of both the substrate peptide (1191.6810 Da) and the N-terminal product peptide (617.3451 Da). The percentage of substrate turnover (product peak integral/(product peak integral + substrate peak integral)*100) was calculated in Microsoft Excel. The experiments were performed in independent replicates (n = 2-4; mean ± standard deviation, SD). IC_{50} curves were processed using non-linear regression using GraphPad Prism 9.3.1 (GraphPad Software Inc., La Jolla, CA).

iii. Pilot Amide Reaction Array Results (Figure 3a).

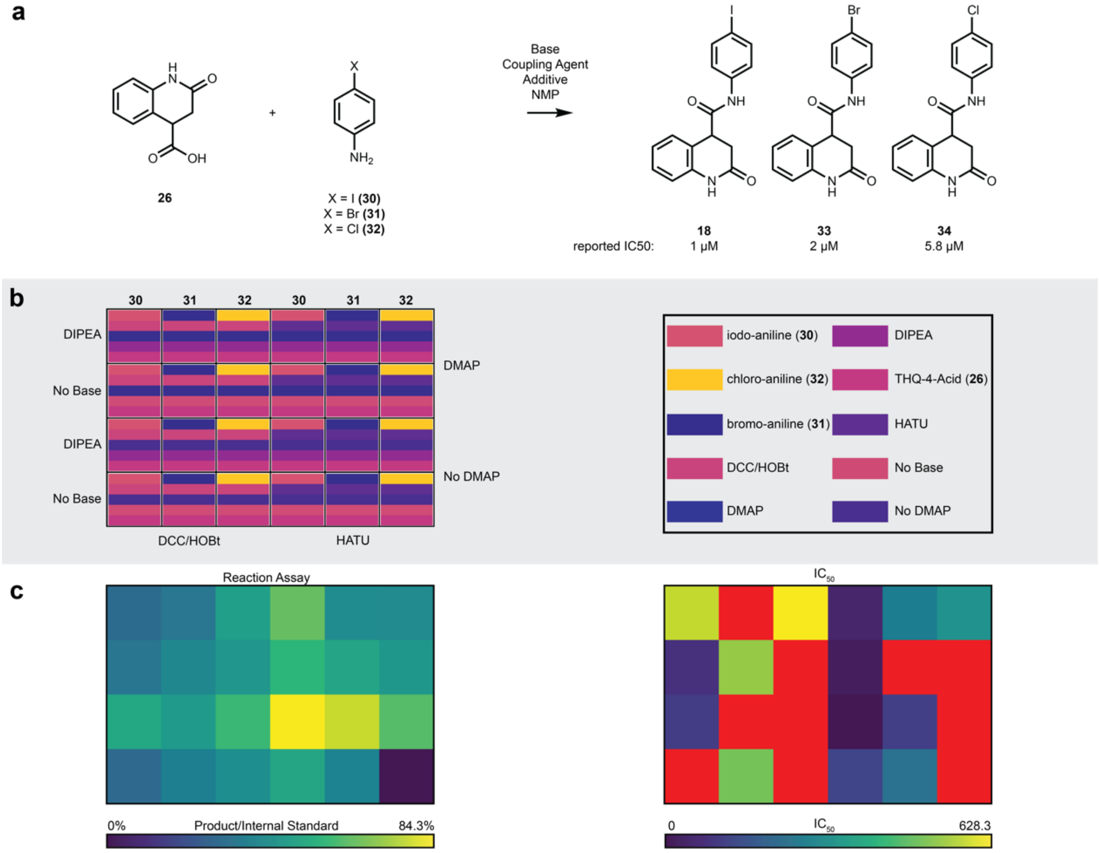

Supplementary Figure 21. a) pilot amide reaction array design to recreate known inhibitors from the literature. b) reaction array layout as designed by phactor™. c) Reaction array results PROD/IS and IC_{50} validating the direct-to-biology procedure. Red cells indicate undetermined IC_{50} values from the assay.

iv. 1,280-well ultraHTE direct-to-biology Procedure. 80 amines (mainly anilines, Supplementary Figure 23, 1 eq.), **26** (1.1 eq.), DIPEA (2 eq.), HATU (1.1 eq.), DCC (1.1 eq.), HOBt (1.1 eq), and DMAP (.1 eq.) were collected from our lab's chemical inventory and prepared as stock solutions in N-methylpyrrolidine based on calculations performed by phactor™ with significant overhead. Stock solutions were plated in a 384-well stock solution wellplate as mapped by NanoChem and distributed into their respective positions on a 1,536 wellplate with the Mosquito® robot as shown in Supplementary Figure 22. 200 µL of each stock reagent was transferred to the reaction plate with no wash volume, resulting in 1 µL reaction volume (0.1 M). DCC and HOBt were co-dosed, and pure NMP was added for wells containing no base or additive. The remaining eight columns were kept empty to serve as placeholders for z'-index columns in the biology assay. Once all stock reagents were distributed into the reaction plate, the plate was centrifuged, sealed, and allowed to react for 24 hours. Once the reactions were complete, the plate was unsealed and each reaction well was diluted to 10 mM with 9µL of 9:1 DMSO:Water solution. The mosquito® robot was used to distribute the quenched reactions into four biology assay plates and four UPLC-MS plates containing equimolar biphenyl as internal standard. Percent inhibition was calculated for each reaction as described in the general information section above. Reproducibility studies are visualized in Supplementary Figure 26 and biology/chemistry trends are plotted in Supplementary Figures 24 and 25. Overall plate results were visualized in phactor™ as shown in Fig. 3 and Fig. 4 of the main text.

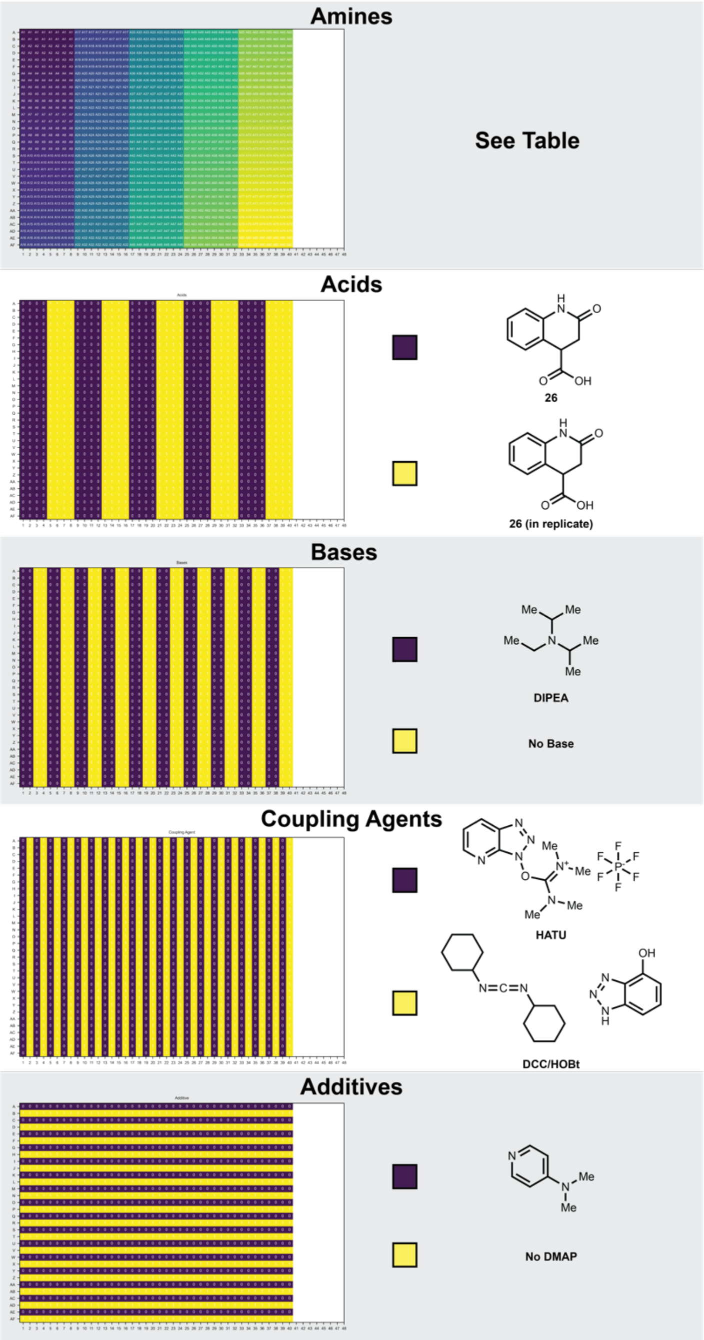

Supplementary Figure 22. ultraHTE direct-to-biology amide coupling reagent distribution as designed by Nanochem and enabled by phactor™. Amines are shown in Supplementary Figure 23.

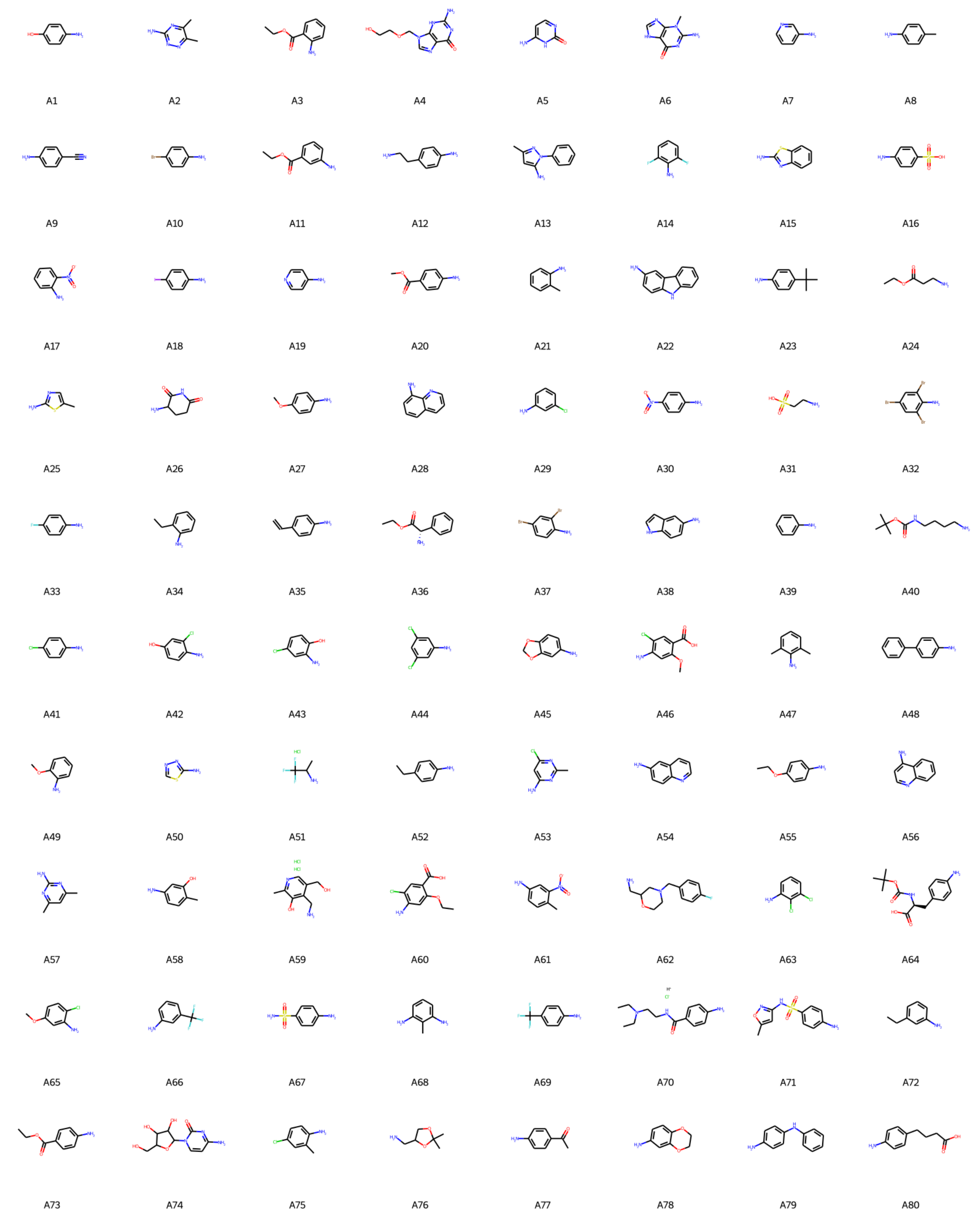

Supplementary Figure 23. Amines used in the amide coupling and distributed according to the design shown in Supplementary Figure 22.

v. 1,280-well ultraHTE direct-to-biology Analysis

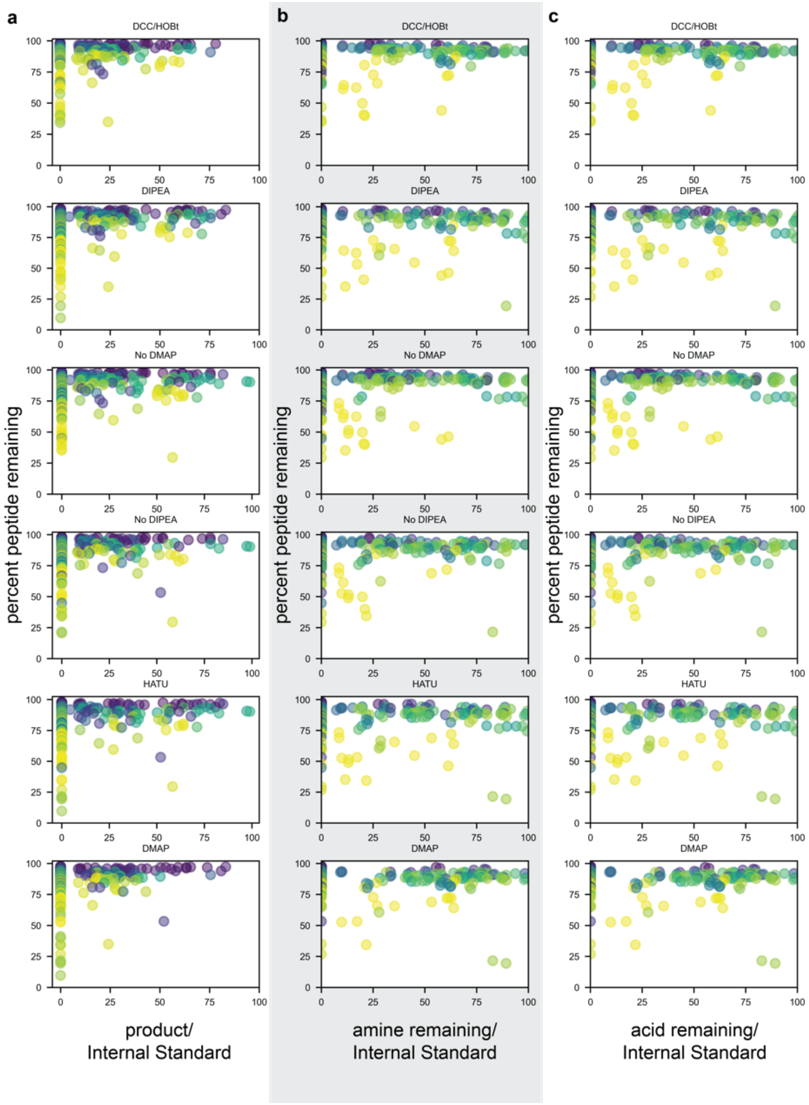

Supplementary Figure 24. PROD/IS vs Substrate turnover scatter plots for each product, amine, and acid for the entirety of the 1,280 well experiment split by reagent (coupling agent, base, and additive).

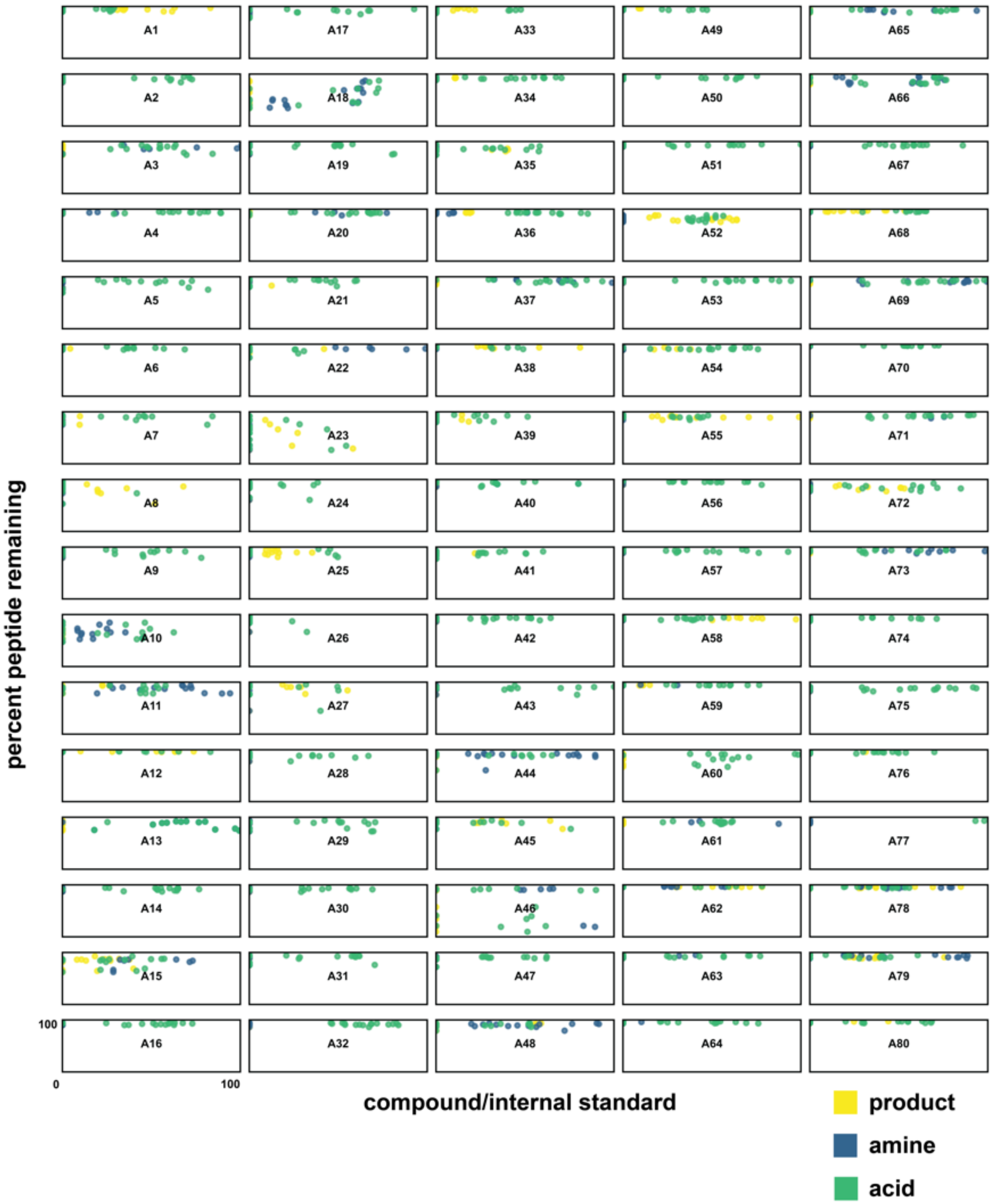

Supplementary Figure 25. PROD/IS vs Substrate turnover scatter plots for each product (16 reactions per desired product).

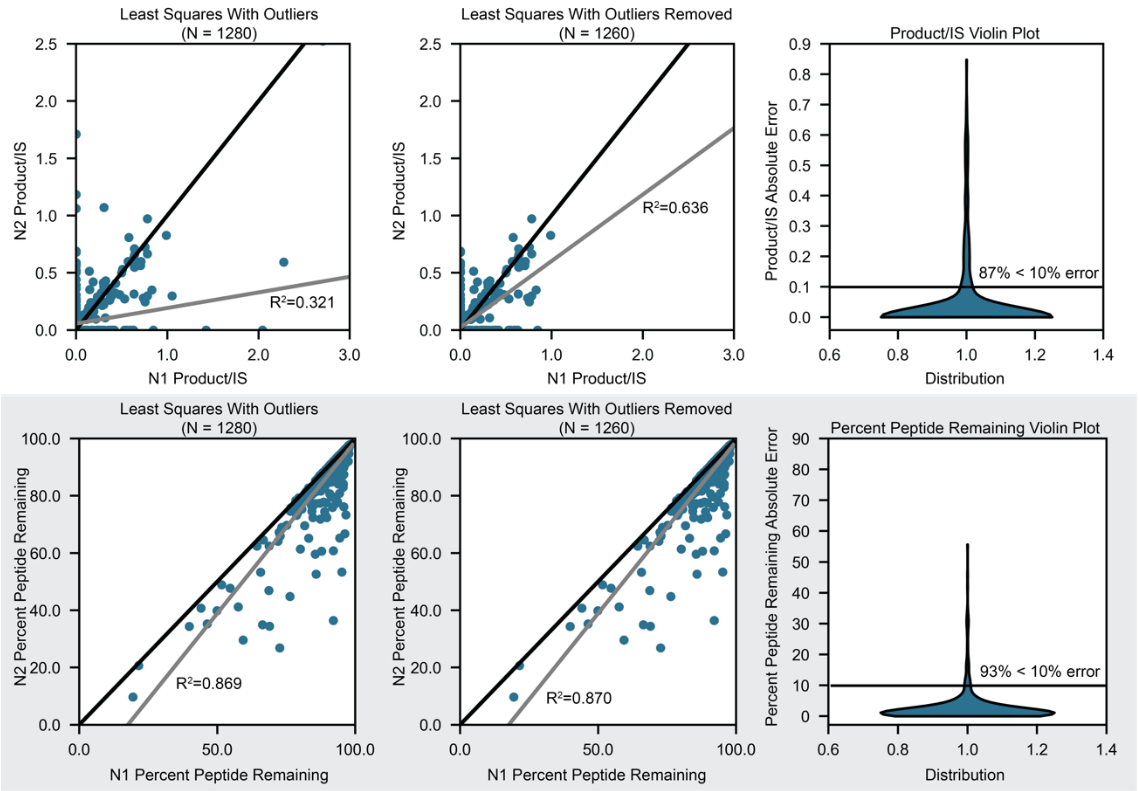

Supplementary Figure 26. Reproducibility studies of the PROD/IS and inhibition assay for the direct-to-biology ultraHTE experiment. Results were found to be highly consistent across the plate.

vi. Preparation of the Carboxylic Acid Pharmacophore Substrate 26 (2-oxo-1,2,3,4-tetrahydroquinoline-4-carboxylic acid)

To a solution of 2-hydroxyquinoline-4-carboxylic acid (1g, 5 mmol) in acetic acid (0.7 M, 7.5 mL) was added 4g of zinc (0.06 mol, 12 eq.) batchwise over one hour. The reaction was stirred under reflux at 70 ºC for two hours. The mixture was then allowed to cool and filtered. The filtrate was then diluted with 10 mL water and placed in a refrigerator overnight. Substrate **26** was then collected as pure crystals (600 mg, 60% isolated yield), dried under high vacuum and stored for future use. Proton and carbon NMR for this compound is displayed in NMR section of the Supplementary Information.

^{1}H NMR (400 MHz, $CDCl_3$) ∂ 10.05 (s, 1H), 7.25 – 7.26 (dd, J = 7.7, 1.5 Hz, 1H), 7.15 – 7.20 (td, *J* = 7.7, 1.5 Hz, 1H), 6.91 – 6.96 (td, J = 7.5, 1.2 Hz, 1H), 6.85 – 6.87 (dd, J = 8.0, 1.2 Hz, 1H), 2.61 – 2.71 (m, 2H). ^{13}C NMR (125 MHz, $CDCl_3$) ∂ 173.81, 169.00, 138.49, 129.42, 128.67, 122.46, 121.17, 115.78, 42.16, 33.07.

vii. Scale up of N-(benzo[d]thiazol-2-yl)-2-oxo-1,2,3,4-tetrahydroquinoline-4-carboxamide (27)

To a solution of **26** (110 mg, 0.55 mmol, 1.1 eq.) in NMP (0.2 M) in a flame-dried 2-dram vial was added HATU (210 mg, 0.55 mmol, 1.1 eq.). This mixture was stirred for five minutes, then to which dimethylaminopyridine (6.1 mg, 50 µM, 0.1 eq.) was added. This mixture was again stirred for five minutes, then to which was added benzo[d]thiazol-2-amine (75 mg, 0.50 mmol, 1 eq.). This final reaction mixture was allowed to stir at room temperature for 24 hours. 8 mg (5%) isolated product was acquired through direct injection of the reaction crude into a reverse-phase preparative HPLC running a water:acetonitrile gradient over 30 minutes. Proton and carbon NMR for this compound is displayed in NMR section of the Supplementary Information. Pure isolate of this compound was subjected to an IC_{50} assay as described in the general information above.

^{1}H NMR (400 MHz, $CDCl_3$) ∂ 10.07 (s, 1H), 7.92 – 7.93 (d, J = 7.9 Hz, 1H), 7.70 – 7.72 (d, J = 8.1 Hz, 1H), 7.46 – 7.47 (d, J = 7.5 Hz, 1H), 7.39 – 7.42 (t, J = 7.6 Hz, 1H), 7.25 – 7.29 (q, J = 7.5, 6.9 Hz, 1H), 6.94 – 6.90 (t, J = 7.5 Hz, 1H), 6.85 – 6.86 (d, J = 8.0 Hz, 1H), 4.15 – 4.17 (dd, J = 6.7, 3.6 Hz, 1H), 2.78 – 2.82 (dd, J = 16.4, 6.6 Hz, 1H), 2.65 – 2.69 (dd, J = 16.4, 3.7 Hz, 1H), 1.21 – 1.24 (d, J = 3.6 Hz, 2H). ^{13}C NMR (125 MHz, $CDCl_3$) ∂ 168.81, 158.09, 148.83, 138.93, 129.07, 128.85, 126.66, 125.87, 124.16, 122.63, 122.18, 121.30, 121.03, 118.17, 116.05, 48.97, 30.57. HRMS (ESI): calculated $C_{17}H_{13}N_3NaO_2S$ [M + Na]: 346.0626, found: 346.0611.

viii. Scale up of N-(4-(tert-butyl)phenyl)-2-oxo-1,2,3,4-tetrahydroquinoline-4-carboxamide (28)

To a solution of **26** (110 mg, 0.55 mmol, 1.1 eq.) in NMP (0.2 M) in a flame-dried 2-dram vial was added HATU (210 mg, 0.55 mmol, 1.1 eq.). This mixture was stirred for five minutes, then to which dimethylaminopyridine (6.1 mg, 50 µM, 0.1 eq.) was added. This mixture was again stirred for five minutes, then to which was added 4-(tert-butyl)aniline (75 mg, 0.50 mmol, 1 eq.). This final reaction mixture was allowed to stir at room temperature for 24 hours. 31 mg (19%) isolated product was acquired through direct injection of the reaction crude into a reverse-phase preparative HPLC running a water:acetonitrile gradient over 30 minutes. Proton and carbon NMR for this compound is displayed in NMR section of the Supplementary Information. Pure isolate of this compound was subjected to an IC_{50} assay as described in the general information above.

^{1}H NMR (400 MHz, $CDCl_3$) ∂ 10.14 (s, 1H), 10.00 (s, 1H), 7.46 – 7.48 (m, 2H), 7.34 – 7.36 (d, J = 7.6 Hz, 1H), 7.27 – 7.29 (m, 2H), 7.13 – 7.21 (m, 1H), 6.89 – 6.92 (t, J = 7.5 Hz, 1H), 6.83 – 6.85 (d, J = 8.0 Hz, 1H), 3.95 – 3.97 (d, J = 6.0 Hz, 1H), 2.69 – 2.73 (m, 1H), 2.58 – 2.62 (dd, J = 16.5 Hz, 4.3 Hz, 1H), 1.22 (s, 9H). ^{13}C NMR (125 MHz, $CDCl_3$) ∂ 170.80, 169.16, 146.29, 138.85, 136.71, 128.54, 128.15, 125.77, 122.43, 122.13, 119.46, 115.86, 43.61, 34.43, 33.42, 31.11. HRMS (ESI): calculated $C_{20}H_{22}N_2NaO_2$ [M + Na]: 345.1579, found: 345.1568.

ix. Scale up of 2-oxo-N-(p-tolyl)-1,2,3,4-tetrahydroquinoline-4-carboxamide (29)

To a solution of **26** (110 mg, 0.55 mmol, 1.1 eq.) in NMP (0.2 M) in a flame-dried 2-dram vial was added HATU (210 mg, 0.55 mmol, 1.1 eq.). This mixture was stirred for five minutes, then to which dimethylaminopyridine (6.1 mg, 50 µM, 0.1 eq.) was added. This mixture was again stirred for five minutes, then to which was added *p*-toluidine (54 mg, 0.50 mmol, 1 eq.). This final reaction mixture was allowed to stir at room temperature for 24 hours. 11 mg (8%) isolated product was acquired through direct injection of the reaction crude into a reverse-phase preparative HPLC running a water:acetonitrile gradient over 30 minutes. Proton NMR for this compound is displayed in NMR section of the Supplementary Information. Pure isolate of this compound was subjected to an IC_{50} assay as described in the general information above.

^{1}H NMR (400 MHz, $CDCl_3$) ∂ 10.10 (s, 1H), 9.99 (s, 1H), 7.43 – 7.45 (d, J = 8.1 Hz, 2H), 7.34 – 7.36 (d, J = 7.6 Hz, 1H), 7.13 – 7.18 (t, J = 7.7 Hz, 1H), 7.07 – 7.08 (d, J = 8.1 Hz, 2H), 6.89 – 6.92 (t, J = 7.5 Hz, 1H), 6.83 – 6.85 (d, J = 7.9 Hz, 1H), 3.94 – 3.97 (t, J = 5.7 Hz, 1H), 2.69 – 2.73 (dd, J = 16.3, 6.5 Hz, 1H), 2.58 – 2.62 (dd, J = 16.3, 4.8 Hz, 1H), 2.22 (s, 3H). ^{13}C NMR (125 MHz, $CDCl_3$) ∂ 170.81, 169.32, 138.77, 136.66, 133.00, 129.57, 128.61, 128.20, 122.54, 122.10, 119.76, 115.91, 43.60, 33.40, 20.83. HRMS (ESI): calculated $C_{17}H_{16}N_2NaO_2$ [M + Na]: 303.1109, found: 303.1102.

e. NMR Spectra

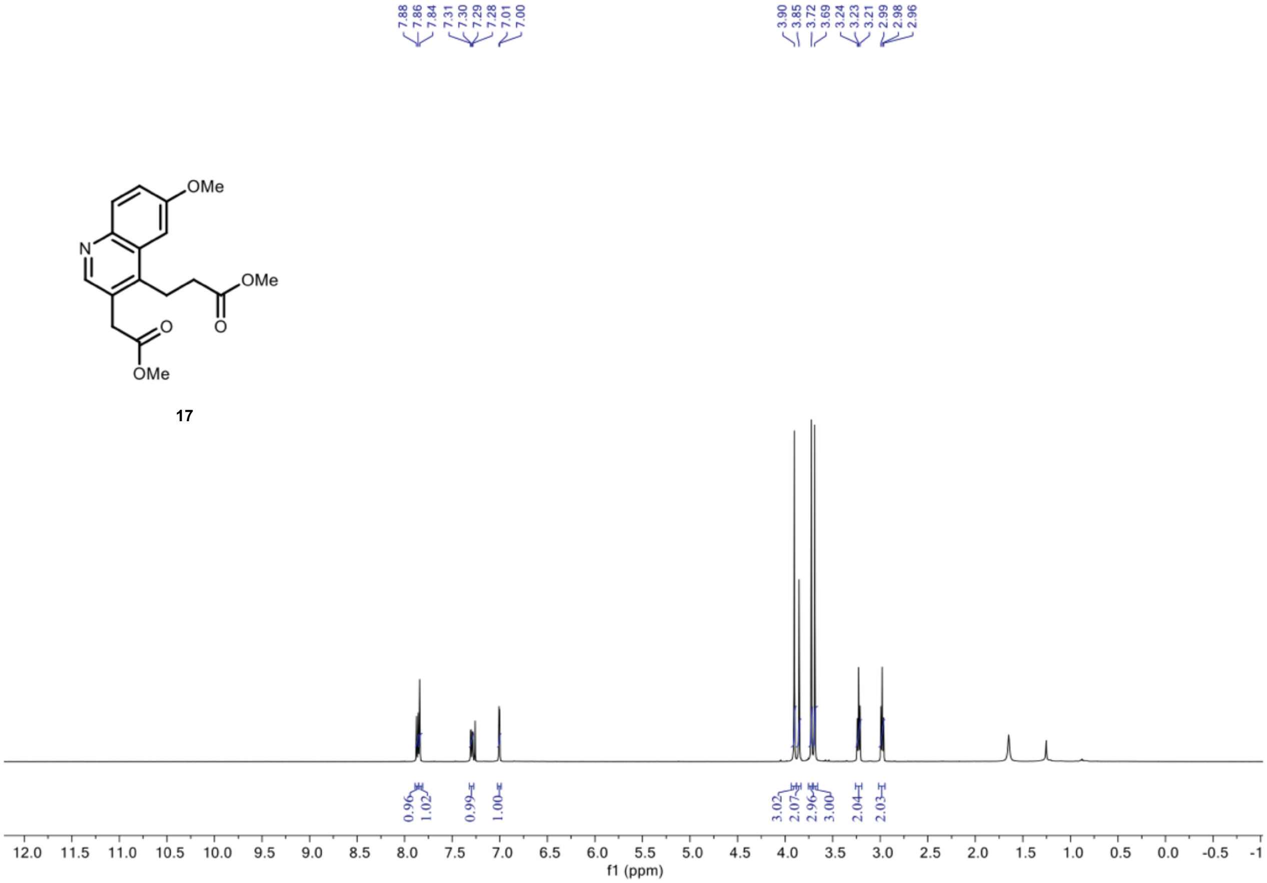

Supplementary Figure 27. Proton NMR of methyl 3-(6-methoxy-3-(2-methoxy-2-oxoethyl)quinolin-4-yl)propanoate.

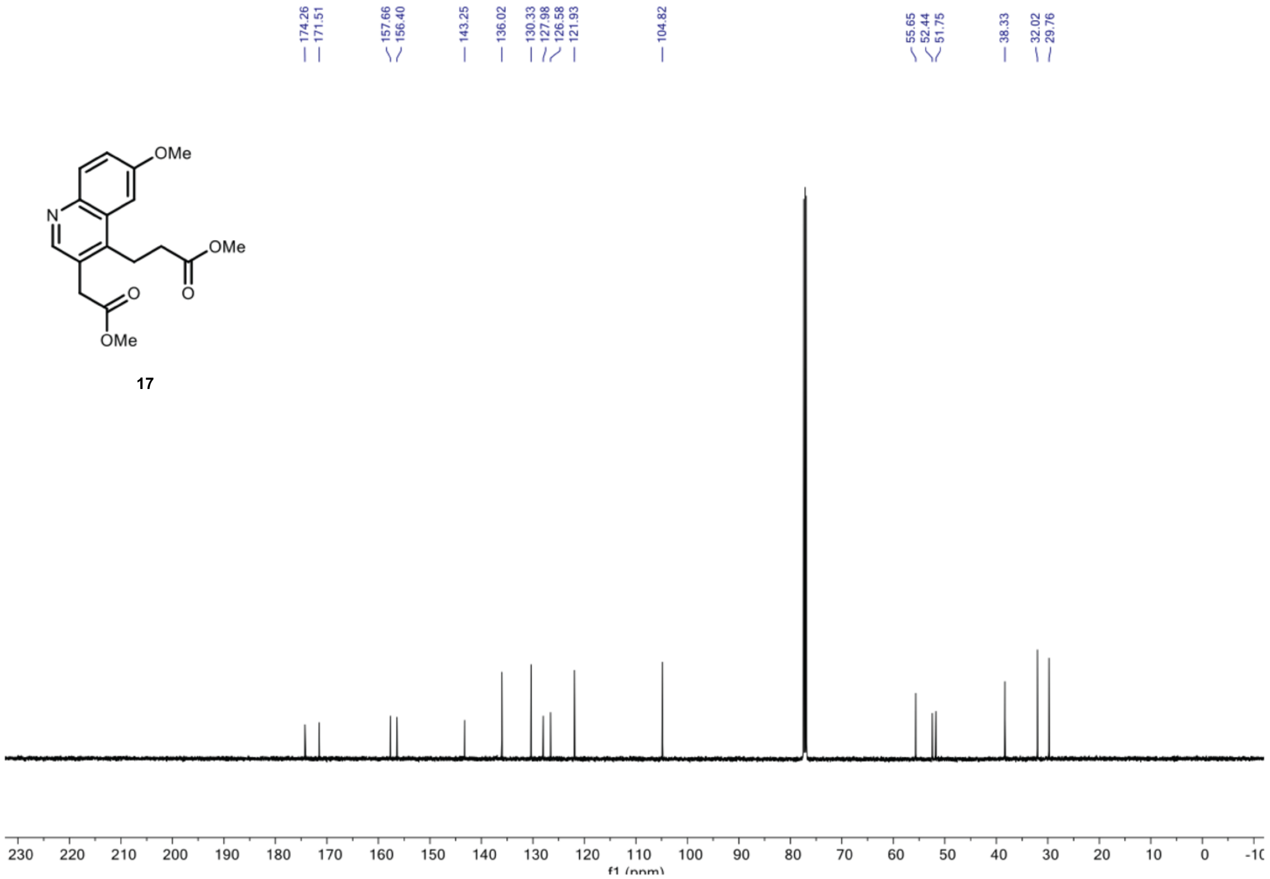

Supplementary Figure 28. Carbon NMR of methyl 3-(6-methoxy-3-(2-methoxy-2-oxoethyl)quinolin-4-yl)propanoate.

1. 2-oxo-1,2,3,4-tetrahydroquinoline-4-carboxylic acid (**26**)

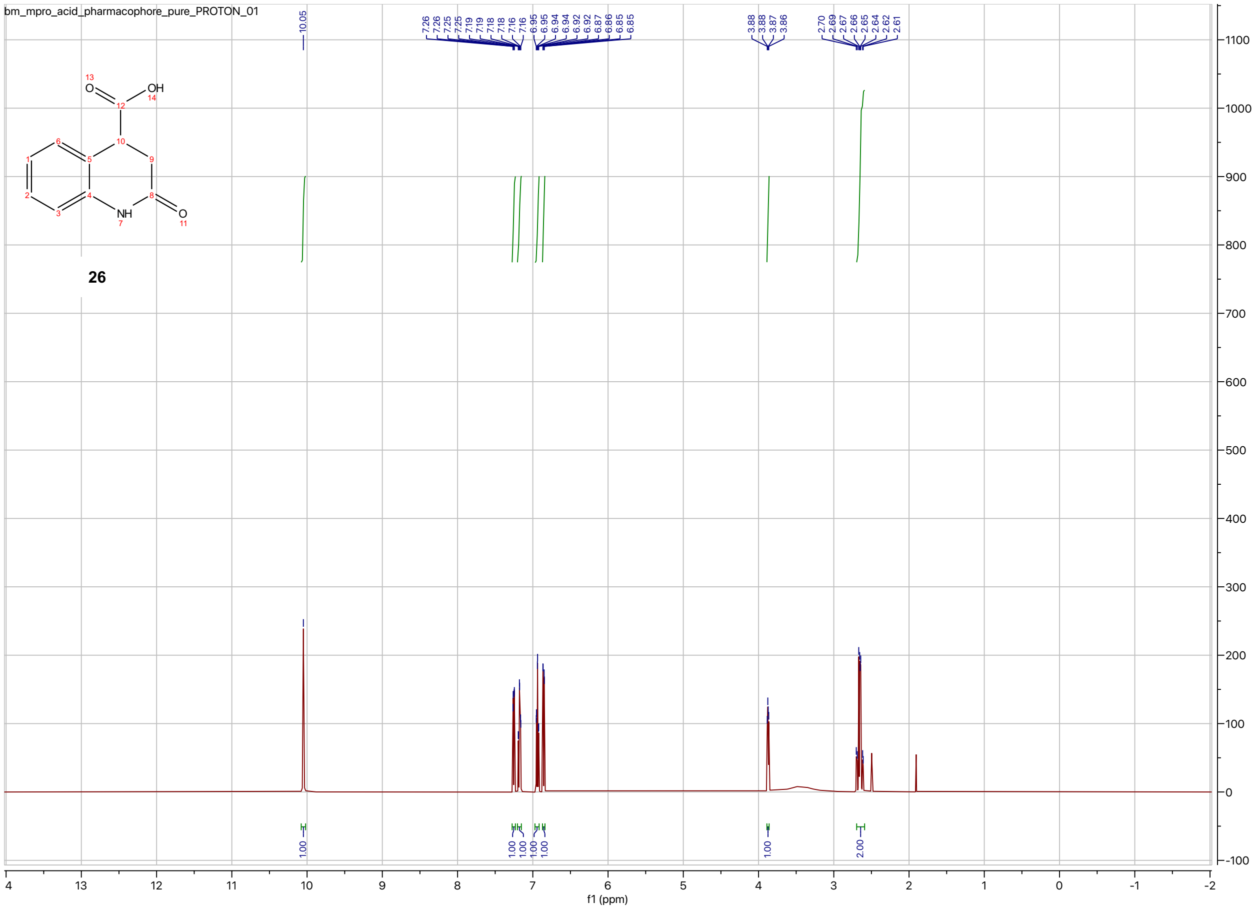

Supplementary Figure 29. Proton NMR of 2-oxo-1,2,3,4-tetrahydroquinoline-4-carboxylic acid.

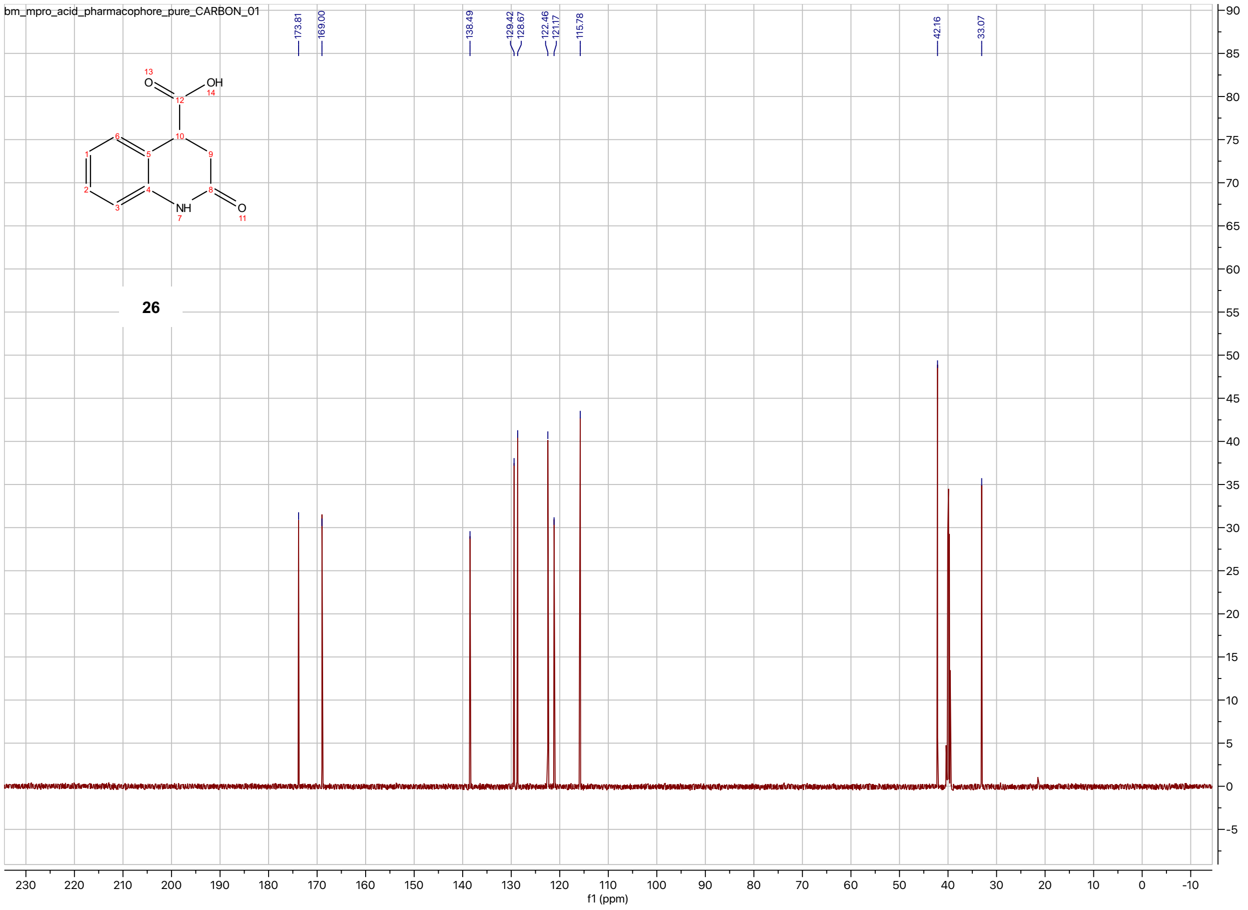

Supplementary Figure 30. Carbon NMR of 2-oxo-1,2,3,4-tetrahydroquinoline-4-carboxylic acid.

2. N-(benzo[d]thiazol-2-yl)-2-oxo-1,2,3,4-tetrahydroquinoline-4-carboxamide (**27**)

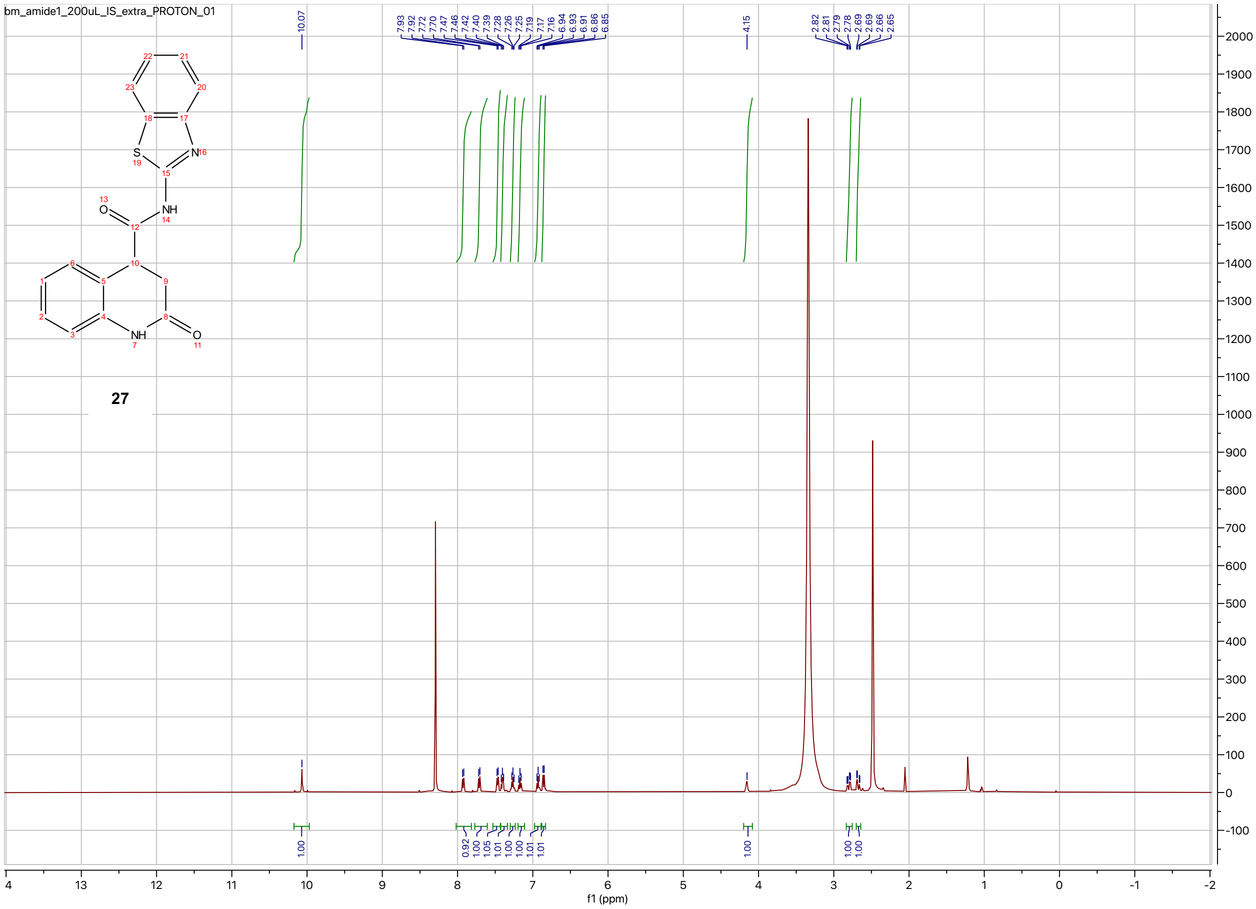

Supplementary Figure 31. Proton NMR of N-(benzo[d]thiazol-2-yl)-2-oxo-1,2,3,4-tetrahydroquinoline-4-carboxamide.

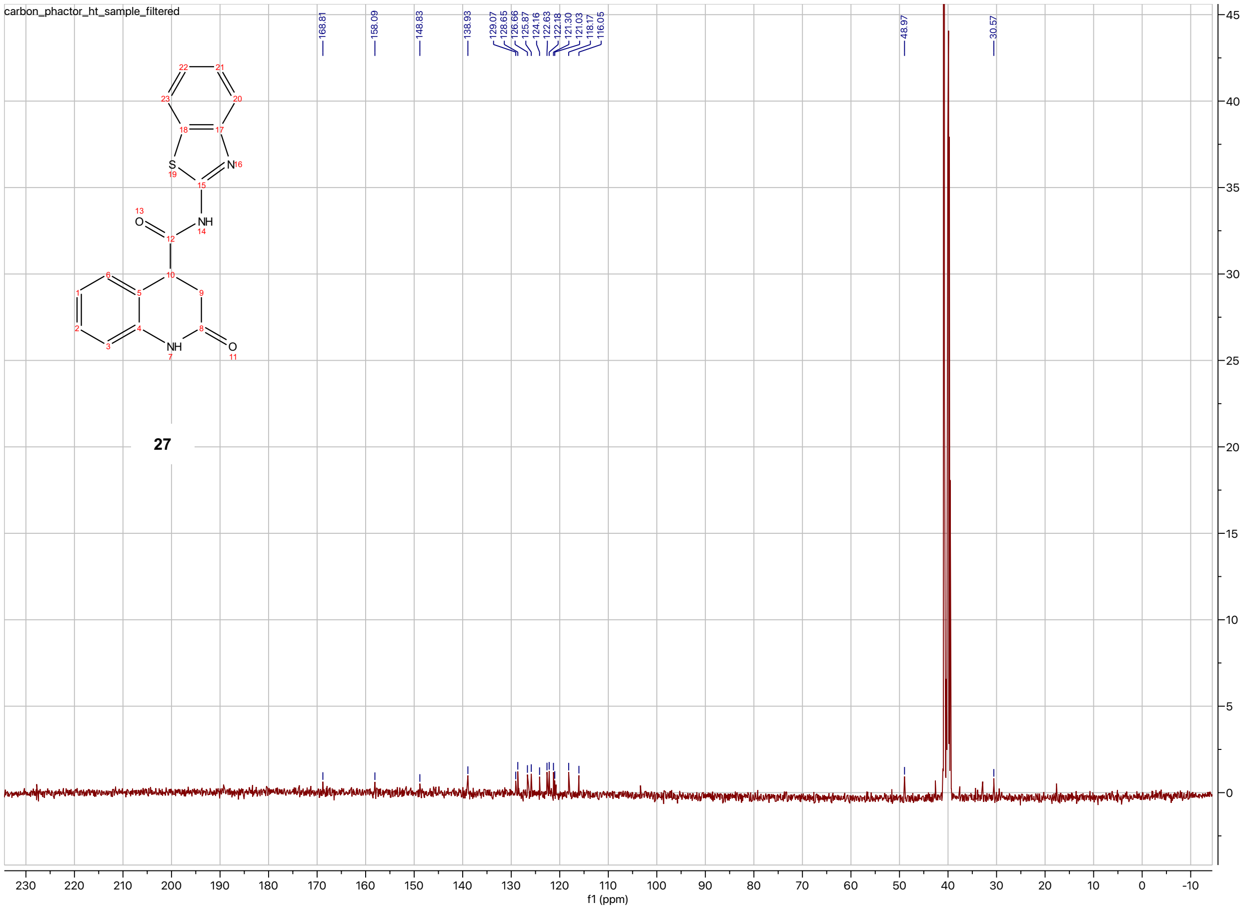

Supplementary Figure 32. Carbon NMR of N-(benzo[d]thiazol-2-yl)-2-oxo-1,2,3,4-tetrahydroquinoline-4-carboxamide.

3. N-(4-(tert-butyl)phenyl)-2-oxo-1,2,3,4-tetrahydroquinoline-4-carboxamide (**28**)

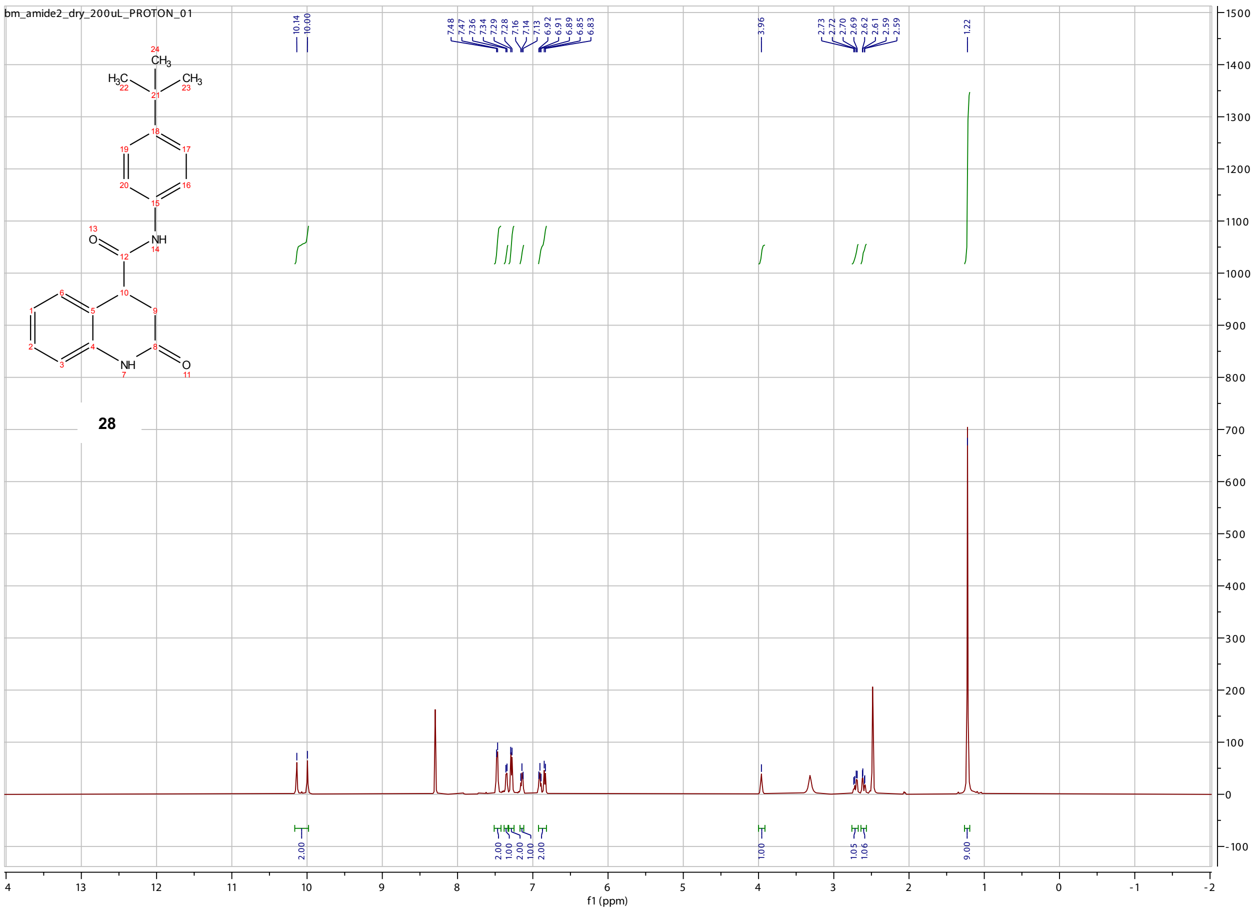

Supplementary Figure 33. Proton NMR of N-(4-(tert-butyl)phenyl)-2-oxo-1,2,3,4-tetrahydroquinoline-4-carboxamide.

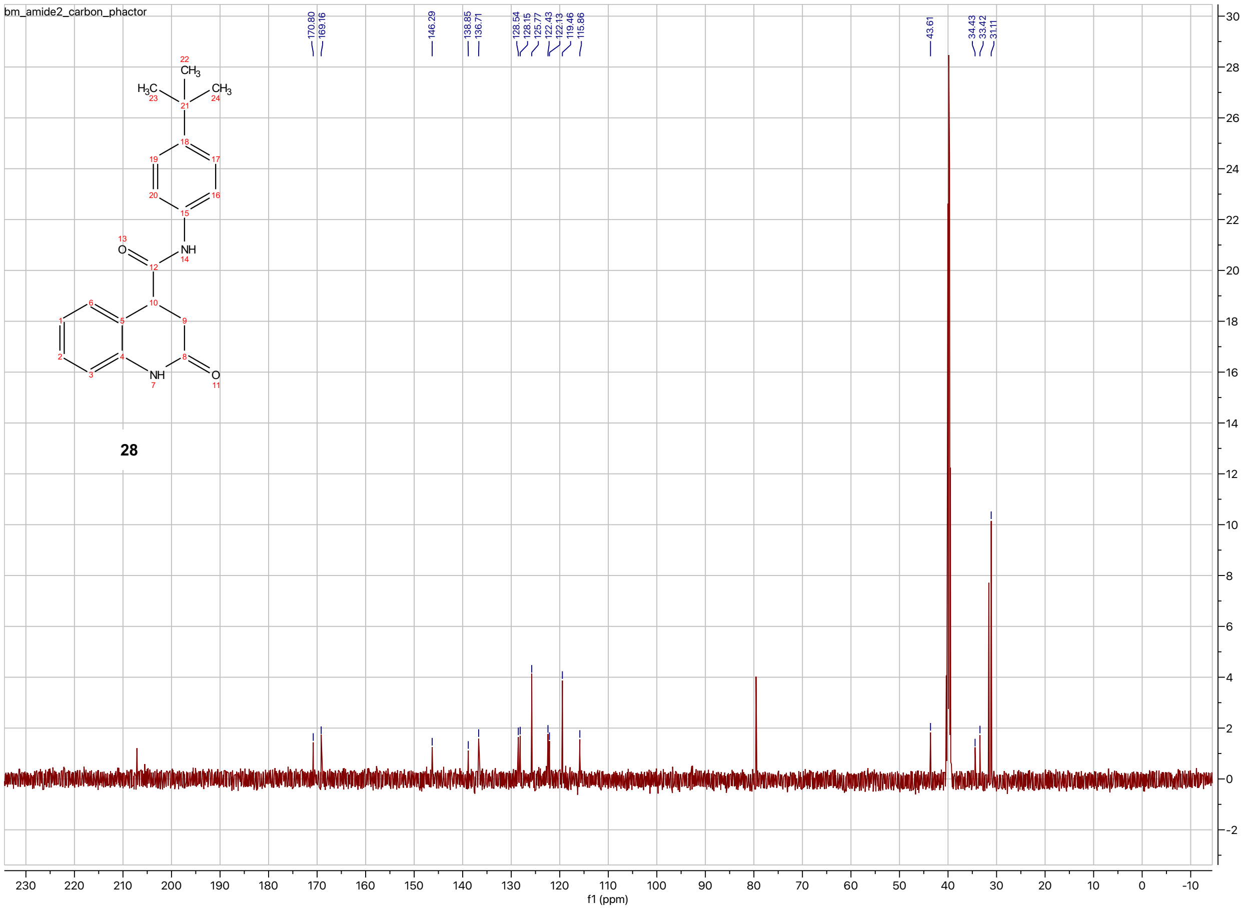

Supplementary Figure 34. Proton NMR of N-(4-(tert-butyl)phenyl)-2-oxo-1,2,3,4-tetrahydroquinoline-4-carboxamide.

4. 2-oxo-N-(p-tolyl)-1,2,3,4-tetrahydroquinoline-4-carboxamide (**29**)

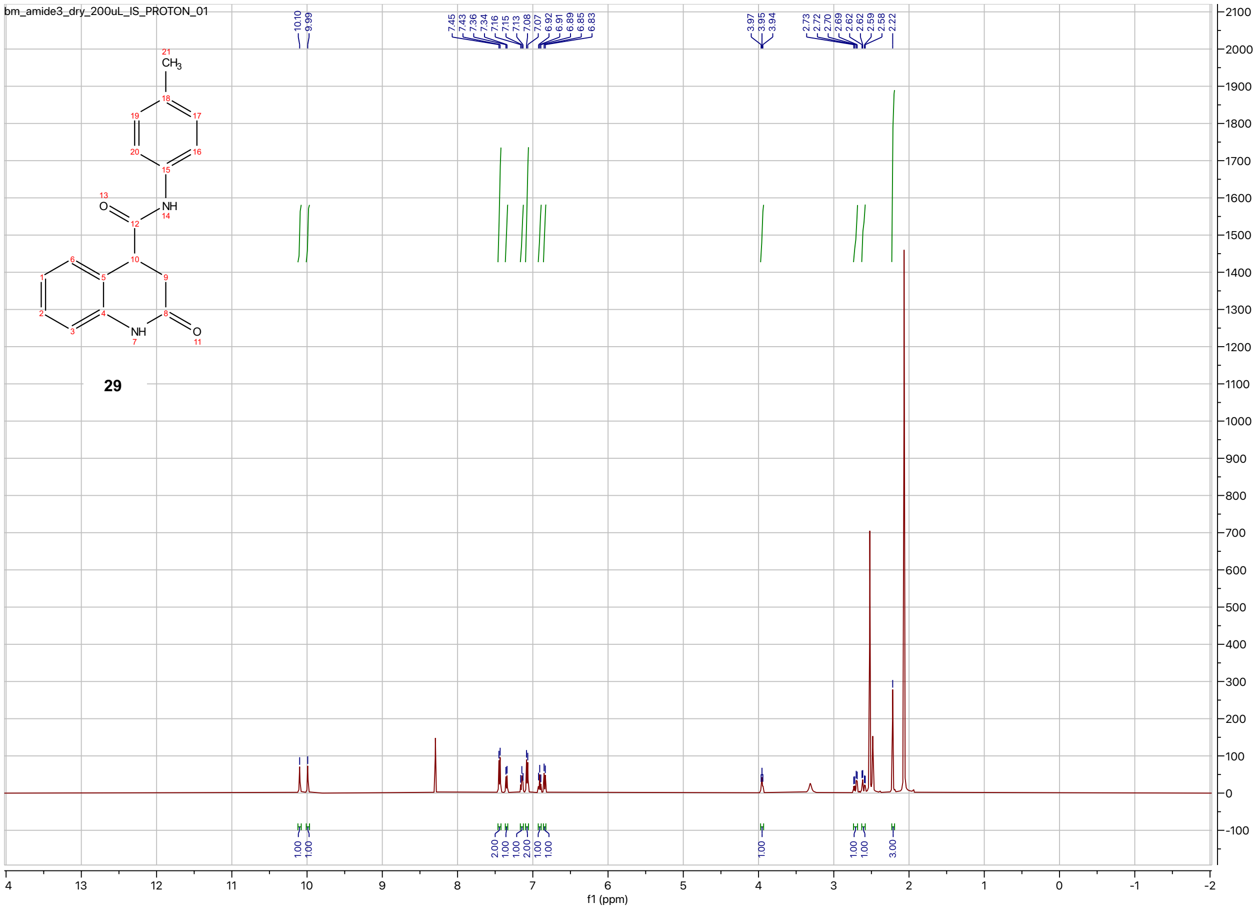

Supplementary Figure 35. Proton NMR of 2-oxo-N-(p-tolyl)-1,2,3,4-tetrahydroquinoline-4-carboxamide.

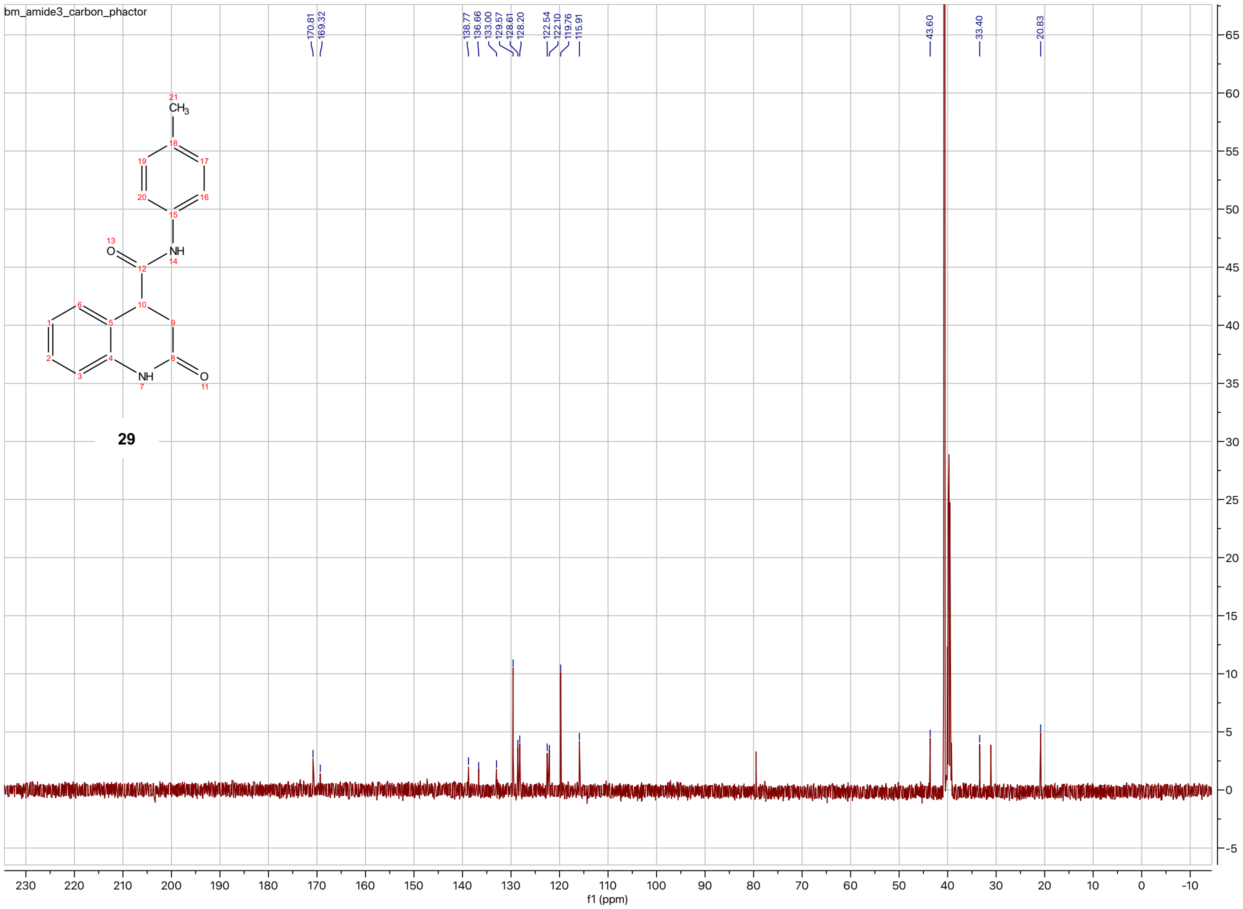

Supplementary Figure 36. Carbon NMR of 2-oxo-N-(p-tolyl)-1,2,3,4-tetrahydroquinoline-4-carboxamide.

Supplementary References

1. Mahjour, B., Shen, Y., Liu, W. & Cernak, T. A map of the amine–carboxylic acid coupling system. *Nature* **580**, 71-75 (2020).
2. Lin, Y. et al. Reinforcing the supply chain of umifenovir and other antiviral drugs with retrosynthetic software. Nature Communications 12, 7327 (2021).
3. Zhang, Z. & Cernak, T. The Formal Cross-Coupling of Amines and Carboxylic Acids to Form sp3–sp3 Carbon–Carbon Bonds. *Angewandte Chemie International Edition* **60**, 27293-27298 (2021).
4. A. Shih-Yuan Lee, L.-S. Lin, Synthesis of allyl ketone via Lewis acid promoted Barbier-type reaction. *Tetrahedron Letters* **41**, 8803-8806 (2000).
5. P. Jones, P. Knochel, Preparation and Reactions of Masked Allylic Organozinc Reagents. *The Journal of Organic Chemistry* **64**, 186-195 (1999).
